# Contact- and Protein Transfer-Dependent Stimulation of Assembly of the Gliding Motility Machinery in *Myxococcus xanthus*

**DOI:** 10.1371/journal.pgen.1005341

**Published:** 2015-07-01

**Authors:** Beata Jakobczak, Daniela Keilberg, Kristin Wuichet, Lotte Søgaard-Andersen

**Affiliations:** Department of Ecophysiology, Max Planck Institute for Terrestrial Microbiology, Marburg, Germany; A*STAR, SINGAPORE

## Abstract

Bacteria engage in contact-dependent activities to coordinate cellular activities that aid their survival. Cells of *Myxococcus xanthus* move over surfaces by means of type IV pili and gliding motility. Upon direct contact, cells physically exchange outer membrane (OM) lipoproteins, and this transfer can rescue motility in mutants lacking lipoproteins required for motility. The mechanism of gliding motility and its stimulation by transferred OM lipoproteins remain poorly characterized. We investigated the function of CglC, GltB, GltA and GltC, all of which are required for gliding. We demonstrate that CglC is an OM lipoprotein, GltB and GltA are integral OM β-barrel proteins, and GltC is a soluble periplasmic protein. GltB and GltA are mutually stabilizing, and both are required to stabilize GltC, whereas CglC accumulate independently of GltB, GltA and GltC. Consistently, purified GltB, GltA and GltC proteins interact in all pair-wise combinations. Using active fluorescently-tagged fusion proteins, we demonstrate that GltB, GltA and GltC are integral components of the gliding motility complex. Incorporation of GltB and GltA into this complex depends on CglC and GltC as well as on the cytoplasmic AglZ protein and the inner membrane protein AglQ, both of which are components of the gliding motility complex. Conversely, incorporation of AglZ and AglQ into the gliding motility complex depends on CglC, GltB, GltA and GltC. Remarkably, physical transfer of the OM lipoprotein CglC to a Δ*cglC* recipient stimulates assembly of the gliding motility complex in the recipient likely by facilitating the OM integration of GltB and GltA. These data provide evidence that the gliding motility complex in *M*. *xanthus* includes OM proteins and suggest that this complex extends from the cytoplasm across the cell envelope to the OM. These data add assembly of gliding motility complexes in *M*. *xanthus* to the growing list of contact-dependent activities in bacteria.

## Author Summary

Motility facilitates a wide variety of processes such as virulence, biofilm formation and development in bacteria. Bacteria have evolved at least three mechanisms for motility on surfaces: swarming motility, twitching motility and gliding motility. Mechanistically, gliding motility is poorly understood. Here, we focused on four proteins in *Myxococcus xanthus* that are essential for gliding. We show that CglC is an outer membrane (OM) lipoprotein, GltB and GltA are integral OM β-barrel proteins, and GltC is a soluble periplasmic protein. GltB, GltA and GltC are components of the gliding motility complex, and CglC likely stimulates the integration of GltB and GltA into the OM. Moreover, CglC, in a cell-cell contact-dependent manner, can be transferred from a *cglC* ^+^ donor to a Δ*cglC* mutant leading to stimulation of gliding motility in the recipient. We show that upon physical transfer of CglC, CglC stimulates the assembly of the gliding motility complex in the recipient. The data presented here adds to the growing list of cell-cell contact-dependent activities in bacteria by demonstrating that gliding motility can be stimulated in a contact-dependent manner by transfer of a protein that stimulates assembly of the gliding motility complexes.

## Introduction

Bacteria interact extensively within and between species to coordinate cellular activities or efficiently compete. These interactions rely on diffusible factors or on direct cell-to-cell contacts [1,2]. Contact-dependent interactions include transfer of DNA or proteins by type IV secretion systems, killing involving the delivery of toxins by the type VI secretion systems, contact-dependent growth inhibition involving two-partner secretion systems, and stimulation of motility in *Myxococcus xanthus* [3–5]. Here, we focused on understanding the contact-dependent mechanism underlying stimulation of gliding motility in *M*. *xanthus*.

Bacterial motility facilitates a wide variety of processes including virulence, biofilm formation and development [6]. Bacteria have at least three mechanisms for motility on surfaces. Rotating flagella propel bacterial cells to bring about translocation [7]. Type IV pili undergo cycles of extension, adhesion to a substratum and retraction to bring about motility [8]. Bacterial cells can also move over surfaces without the aid of flagella and type IV pili by a mechanism referred to as gliding motility [9]. Bacterial flagella are homologous structures present in Gram-positive as well as–negative bacteria, mechanistically fairly well-understood and consists of four parts that together span from the cytoplasm, across the cell envelope to the cell surface [7]. Type IV pili are similarly widespread homologous structures and the machinery underlying type IV pili function also spans from the cytoplasm to the cell surface [8]. Gliding motility is present in Gram-positive as well as in -negative bacteria [9]. In contrast to the homologies observed for flagella and type IV pili systems, the machineries for gliding motility are non-homologous suggesting that gliding motility has evolved independently several times [6,9] and mechanistically, gliding motility is poorly understood.

*M*. *xanthus* is a rod-shaped, Gram-negative bacterium that has two genetically distinct motility systems that allow translocation on solid surfaces in the direction of the long axis of a cell [10]. One system depends on type IV and is often referred to as S-motility [10,11]. Gliding motility, often referred to as A-motility in *M*. *xanthus*, allows movement of single cells [10]. The force for gliding is generated along the cell length [12–14]. Consistently, several proteins that are required for gliding motility have been shown using fluorescently-tagged fusion proteins to localize to clusters that are distributed along the length of the ventral side of the cell [13,15–18]. Traction force is generated at the position of these clusters supporting the notion that these clusters represent gliding motility complexes [13]. The gliding motility complexes assemble at the leading cell pole, remain stationary with respect to the substratum as a cell is moving, and disassemble as they approach the lagging cell pole. During gliding *M*. *xanthus* cells deposit slime trails of unknown composition and the motility complex have been suggested to attach to the substratum *via* the slime [19].

**Fig 1:**
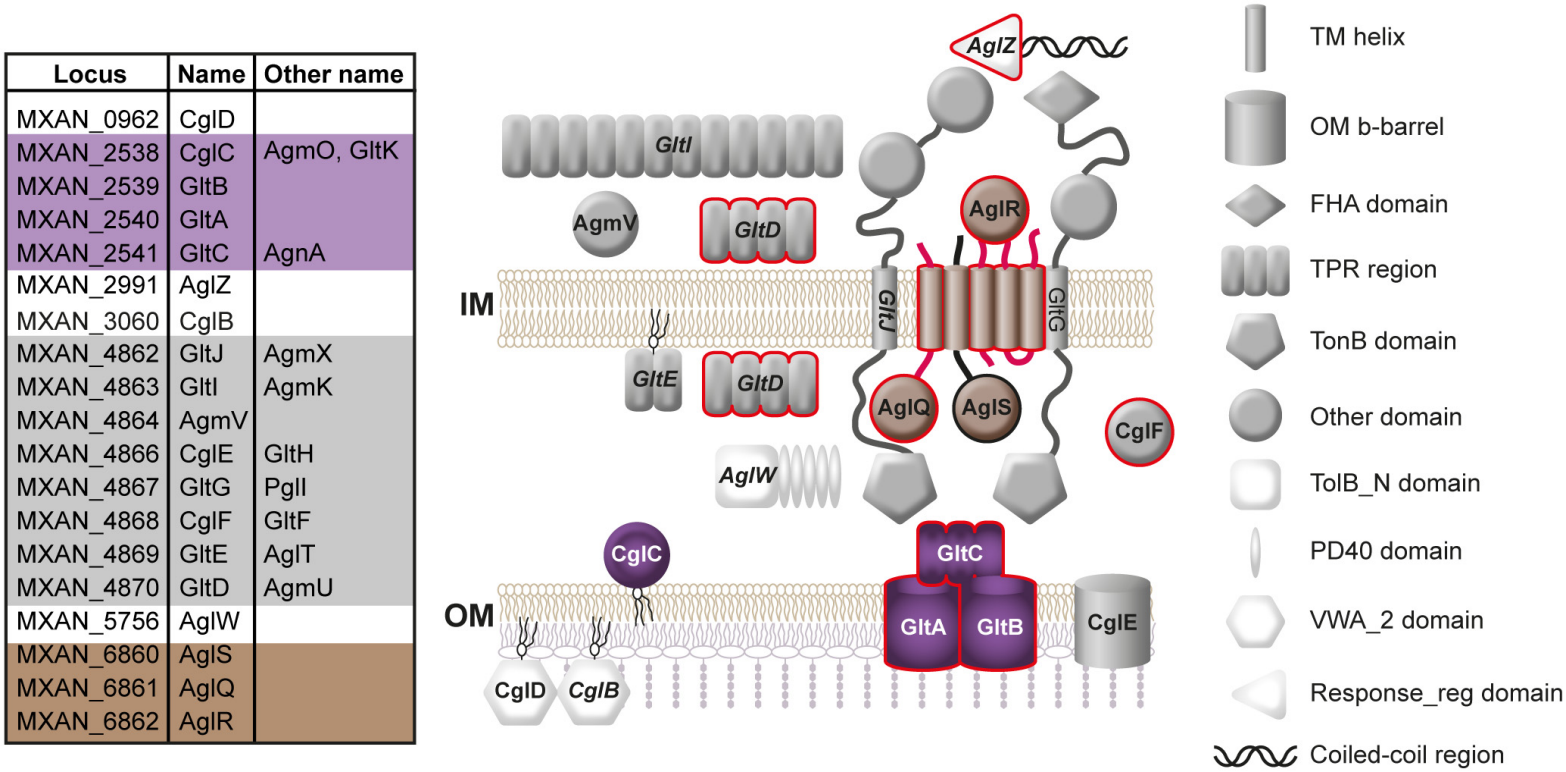
Subcellular localization of proteins required for gliding motility. Synonyms for proteins required for gliding motility are indicated on the left. Proteins shown on a grey, brown or purple background in the left panel and in grey, brown or purple in the right panel are encoded together in the genome [17]; proteins in white are not encoded near other proteins shown here. Proteins outlined in red have been shown by fluorescence microscopy to localize in clusters along the cell body [here; [15–18]]; proteins that interact based on pull down experiments using *M*. *xanthus* cell extracts are indicated in italics [15]. CglB is an OM lipoprotein [29] and CglD is predicted to be an OM lipoprotein [20]. It is not known if they face towards the periplasm or are exposed on the cell surface. They are shown on the cell surface because they contain a von Willebrand domain (VWA_2), which is often involved in cell adhesion [30].

Numerous proteins involved in gliding motility have been described [13,17,18,20–22]. Genetic and cytological evidence suggests that gliding motility is driven by a protein complex that spans part or all of the cell envelope. This complex includes the AglR, AglQ and AglS proteins, which are homologs of MotA/TolQ/ExbB (AglR) and MotB/TolR/ExbD (AglQ and AglS) and form a proton channel in the inner membrane (IM) [13,18]. AglQ and AglR have been shown directly to localize to the clusters of motility complexes [13,23] (Fig 1). Additional proteins that localize to the cytoplasm, IM, periplasm or outer membrane (OM) have been suggested to be components of the gliding motility complex. These proteins include the 11 GltA-K proteins that are encoded by two gene clusters (Fig 1) and among which the eight GltA-H are paralogs of the NfsA-H proteins that are important for spore formation [17,24–27]. The NfsA-H proteins have been suggested to form a complex that spans the cell envelope and with protein localizing to the IM, periplasm and OM. Similarly, the GltA-K proteins have been suggested to form a complex that would span from the cytoplasm to the OM [17]. Several of the proteins required for gliding motility have been shown to interact based on pull down experiments [15,17] (Fig 1). However, only the cytoplasmic AglZ [16], CglF (GltF), which is reported to be a periplasmic as well as an IM protein, and GltD, which is reported to localize to the cytoplasm and periplasm, have been shown to localize to the clusters of motility complexes along the cell length [15,17] (Fig 1). GltD and AglR have also been reported to localize to a rotating helical structure [18,23]. Accordingly, two models for the gliding machinery in *M*. *xanthus* have been proposed. In both models, motility complexes are distributed along the length of the ventral side of the cell. In the focal adhesion model, the motility complex spans from the cytoplasm over the IM, periplasm, OM to the cell surface where it connects to the substratum *via* the slime and generate traction force [13,17,19]. In the helical rotor model, the motility complex spans the IM and periplasm and move on a rotating helix. These complexes are hypothesized to slow down close to the substratum in that way forming clusters and causing a deformation of the cell surface. These deformations, in turn, generate drag forces between the cell and the substratum [18,23]. An analysis of the biophysical properties of cell-substrate interactions during gliding, provided evidence in favor of a focal adhesion model [28]. A prediction from the focal adhesion complex model is that the gliding motility complex includes proteins that localize to the OM.

Even though several proteins that are essential for gliding motility have been predicted or directly shown to localize to the OM [17,29,31,32] (Fig 1), none of these proteins have been shown to be part of the gliding motility complex. Interestingly, several gliding motility mutants including *cglC* mutants can be transiently stimulated to move by gliding upon direct contact with cells that are wild-type (WT) for the corresponding genes [33]. This process, termed stimulation, depends on the contact-dependent physical transfer of OM lipoproteins between cells [29,34–36] in a process that may involve fusion of the OMs of donor and recipient [36,37]. Currently, it is not known how transfer of lipoproteins required for gliding results in stimulation of gliding in *M*. *xanthus*. Interestingly, in the type IV pili system of *M*. *xanthus*, lack of the Tgl lipoprotein results in a defect in type IV pili-dependent motility [33]. Type IV pili-dependent motility can be transiently restored in a *tgl* mutant by the contact-dependent physical transfer of Tgl from a *tgl* ^+^ donor [29,33]. In the recipient cells, transferred Tgl stimulates multimer formation by the PilQ secretin in the OM thereby allowing assembly of the type IV pili machinery [29,38,39].

Here, we identify the first OM components of the gliding machinery. We demonstrate that GltB and GltA are OM β-barrel proteins that form a subcomplex with the periplasmic GltC protein and that this subcomplex is an integral part of the gliding motility complex. Moreover, our data suggest that the OM lipoprotein CglC stimulates gliding by facilitating the OM integration of GltB and GltA. Also, our data demonstrate that assembly of the gliding motility complex depends on CglC, GltB, GltA and GltC as well as on AglZ in the cytoplasm and AglQ in the IM suggesting a non-hierarchical assembly pathway for the motility complex. Intriguingly, upon stimulation of a Δ*cglC* recipient by a *cglC* ^+^ donor, CglC instigates the assembly of the gliding motility complex in the recipient likely by facilitating the integration of GltB and GltA into the OM in the recipient. Therefore, transferred CglC directly stimulates assembly of the gliding motility complex.

## Results

### CglC, GltB, GltA and GltC are essential for gliding motility

**Fig 2:**
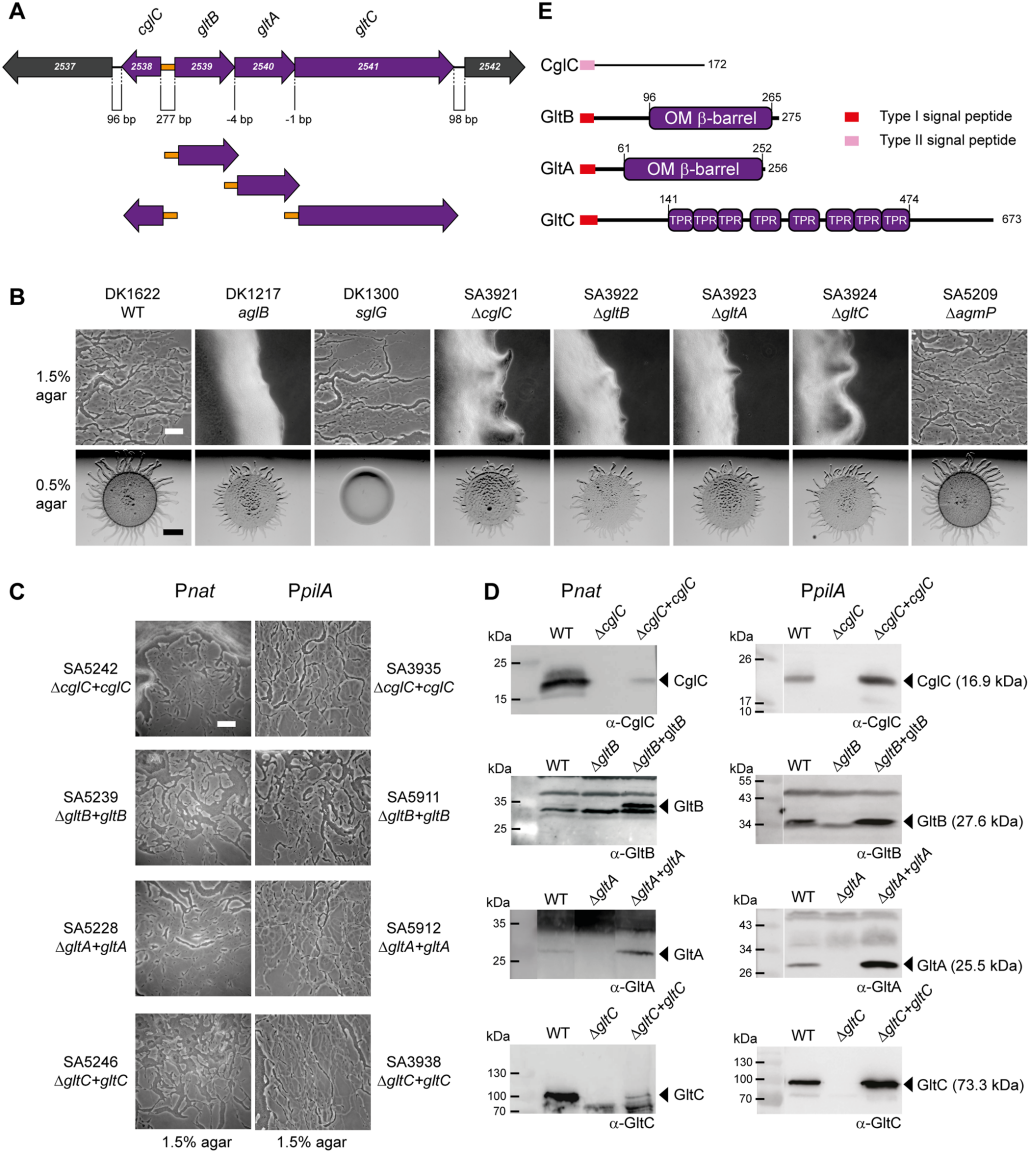
CglC, GltB, GltA and GltC are required for gliding motility. (A) Genetic organization of *cglC*, *gltB*, *gltA* and *gltC* region. Numbers below diagram indicates distances between start and stop codons of flanking genes. Orange box indicates the intergenic region containing the divergent *cglC* and *gltBAC* promoters. The four complementation constructs with the native promoter (orange) are indicated below. (B, C) Motility phenotypes of the indicated mutants (B) and complementation strains (C). Cells were incubated at 32°C for 24 h on 0.5% and 1.5% agar to score for type IV pili-dependent and gliding motility, respectively. In-frame deletion mutants are shown in (B) and the corresponding complementation strains are shown in (C). In the rows labeled P_*nat*_ and P_*pilA*_, the complementing genes were expressed from the native promoter (marked orange in panel A) and the *pilA* promoter, respectively. Scale bar, 50μm on 1.5% agar and 2 mm on 0.5% agar. (D) Accumulation of CglC, GltB, GltA and GltC. Total cell lysates from exponentially growing cultures were separated by SDS-PAGE (proteins from 7×10^7^ cells loaded per lane) and analyzed by immunoblotting using specific antibodies as indicated. In the left and right panels, the complementation strains express the relevant gene from the native and the *pilA* promoter, respectively. Arrowheads indicate the relevant protein with the calculated molecular mass without signal peptides in brackets. Molecular mass markers are indicated in the leftmost lane of the two panels. Note that GltC has a calculated molecular mass of 73.3 kDa but is consistently observed to run as a higher molecular mass protein by SDS-PAGE. (E) Domain structure of CglC, GltB, GltA and GltC.

While studying the RomR response regulator that is required for full function of both motility systems in *M*. *xanthus* [40,41], we isolated miniHimar insertion mutations that caused defects in gliding motility. Two of these insertions mapped to MXAN_2540 and MXAN_2541 (Fig 2A). Previously, MXAN_2538, MXAN_2541 and MXAN_2542 were identified in transposon mutagenesis screens and suggested to be important for gliding motility [21,22]. Luciano et al. [17] showed that in-frame deletions of MXAN_2538, MXAN_2539, MXAN_2540 or MXAN_2541 caused gliding motility defects and named these genes *gltK*, *gltB*, *gltA* and *gltC*, respectively (Fig 1). Because MXAN_2538 was recently shown to be identical to *cglC* [20], we investigated these genes in more detail to gain insight into how CglC may stimulate gliding motility.

To verify the results of the transposon mutagenesis screen and previously published data, in-frame deletions in each of the five genes from MXAN_2538-MXAN_2542 were generated in the fully motile WT strain DK1622. Motility assays using DK1622 and verified mutants for gliding (DK1217) and type IV pili-dependent motility (DK1300) as controls, demonstrated that WT as well as the five in-frame deletion mutants formed the rafts characteristic of type IV pili-dependent motility on 0.5% agar, which is favorable to type IV pili-dependent motility only [42], whereas DK1300 as expected did not form these rafts (Fig 2B). On 1.5% agar, which is favorable to gliding motility only, DK1622 displayed the single cells and slime trails characteristic of gliding motility at the edge of the colony. In contrast, neither the DK1217 control strain nor the mutants with in-frame deletions of MXAN_2538, MXAN_2539, MXAN_2540 or MXAN_2541 did. The in-frame deletion of MXAN_2542 did not have any effect on gliding motility. In agreement with these findings, it was recently reported using a Tn*5* transposon insertion in MXAN_2542 that this gene is not essential for gliding [20]. Consistently, we found that MXAN_2539, MXAN_2540 or MXAN_2541 were transcribed as a single polycistronic mRNA that did not include MXAN_2542 using reverse transcription-PCR on total RNA isolated from exponentially growing WT cells (S1 Fig). Henceforth, the *cglC*, *gltB*, *gltA* and *gltC* nomenclature is used for MXAN_2538-_2541.

CglC, GltB, GltA and GltC bear the hallmarks indicative of cell envelope localization: CglC contains a type II lipoprotein signal peptide followed by a region that does not contain known domains (Fig 2E) in agreement with previous analyses [20]. GltB, GltA and GltC all contain a type I signal peptide. GltB and GltA are paralogs and have a domain homologous to the OM β-barrel domain of OmpA from *Escherichia coli* (S2 Fig) suggesting that GltB and GltA are integral OM proteins. GltC contains multiple TPR repeats that are frequently observed in proteins in multi-protein assemblies. Moreover, the GltB and GltA paralogs NfsB and NfsA are also predicted to be integral OM β-barrel proteins and localize to the OM when expressed in *E*. *coli* [25]. Also, the GltC paralog NfsC associates with the OM when expressed in *E*. *coli* [25].

To confirm that the gliding motility defects caused by the in-frame deletions of *cglC*, *gltB*, *gltA* and *gltC* were due to the lack of the corresponding protein and not a polar effect on downstream genes, we carried out complementation experiments in which *cglC* was cloned downstream of its native promoter and *gltB*, *gltA and gltC* separately cloned downstream of the promoter of the *gltBAC* operon (Fig 2A). The corresponding plasmids were integrated ectopically at the phage Mx8 *attB* site in the relevant mutants. The gliding defect in all four mutants was complemented by the ectopic copy of the corresponding WT gene (Fig 2B and 2C). Immunoblot analyses demonstrated that CglC and GltC accumulated at lower levels in the complementation strains than in WT (Fig 2D). In contrast, GltB and GltA accumulated at higher levels in the complementation strains than in WT despite using the same promoter for expression of *gltB*, *gltA* and *gltC*. In complementation strains in which *cglC*, *gltB*, *gltA* and *gltC* were expressed from the *pilA* promoter full complementation was also observed (Fig 2B and 2C). In these four strains, CglC and GltA accumulated at higher levels than in WT and GltB and GltC accumulated at levels similar to those in WT (Fig 2D). Thus, we conclude that CglC, GltB, GltA and GltC are required for gliding motility.

### CglC, GltB, GltA and GltC localize to the cell envelope

**Fig 3:**
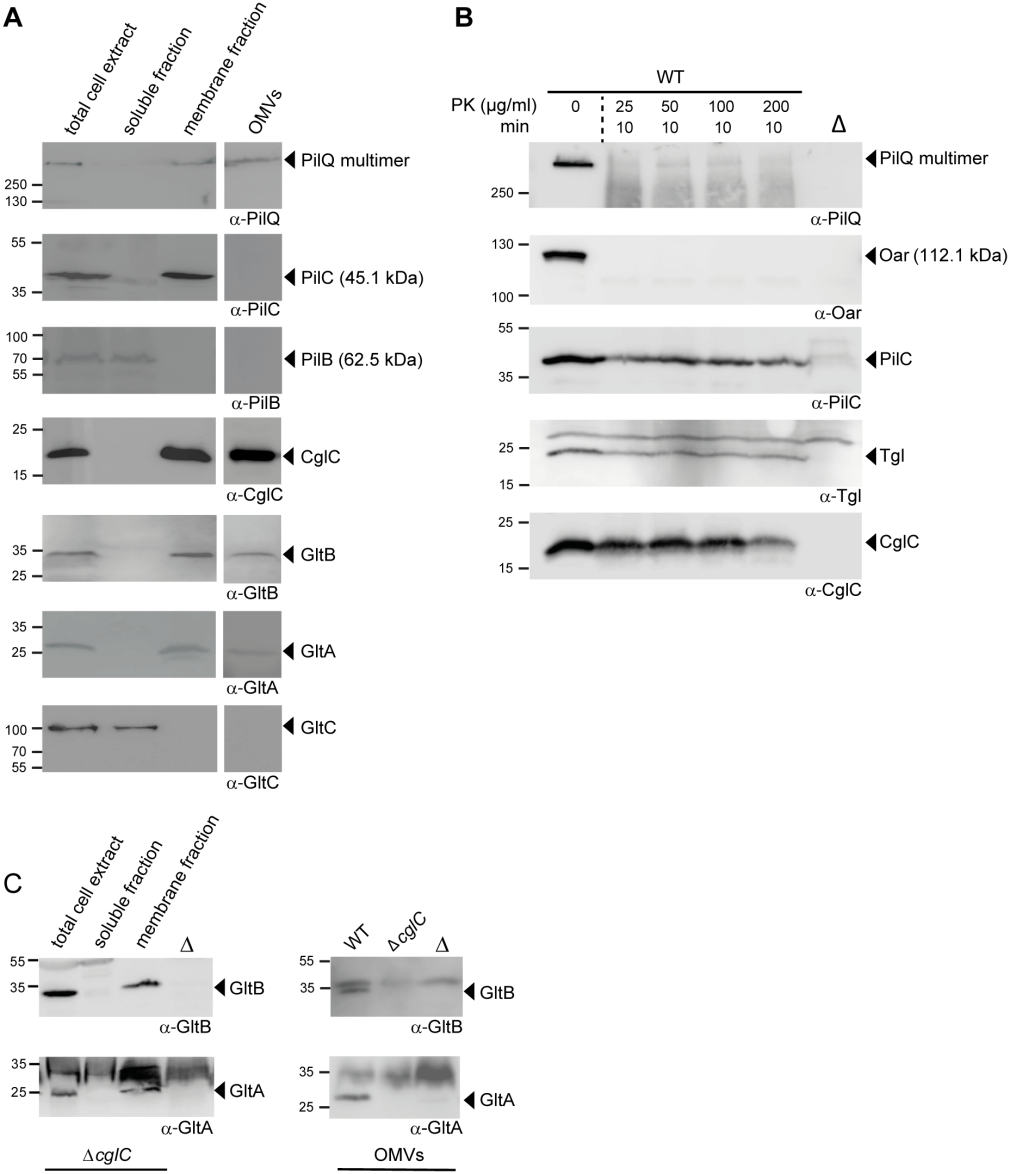
Subcellular localization of CglC, GltB, GltA and GltC. (A) GltB, GltA and GltC localize to the OM and GltC is a soluble protein. Total WT cell extracts were separated into soluble and membrane fractions. Outer membrane vesicles (OMVs) were isolated from the cell free supernatant. Fractions were analyzed by immunoblotting using the indicated antibodies. Molecular mass markers are indicated to the left. The relevant proteins are indicated. (B) CglC is facing towards the periplasm. WT cells were treated with Proteinase K (PK) at the indicated concentrations for 10 min, followed by SDS-PAGE and immunoblotting. Lanes labeled Δ contain total cell extracts from the relevant in-frame deletion mutant. (C) CglC is important for OM incorporation of GltB and GltA. Left panel, total cell extracts from Δ*cglC* cells were separated into soluble and membrane fractions. Right panel, OMVs were isolated as in (A) from the indicated strains. All fraction were analyzed by immunoblotting using the indicated antibodies. Lanes labeled Δ contain total cell extracts from the relevant in-frame deletion mutants.

To determine the subcellular localization of CglC, GltB, GltA and GltC, fractionation experiments were performed in which total cell extract, the soluble fraction enriched for cytoplasmic and periplasmic proteins, the membrane fraction enriched for IM and OM proteins, and OM vesicles (OMVs) were isolated from exponentially growing *M*. *xanthus* cells. As shown in Fig 3A, the control proteins multimeric PilQ in the OM [39], PilC in the IM [43] and PilB in the cytoplasm [44] fractionated as expected, confirming the successful fractionation. CglC, GltB and GltA fractionated with the membrane fraction as well as with OMVs. GltC fractionated with the soluble fraction and was neither detected in the membrane fraction nor in OMVs. In combination with the sequence analyses, we conclude that CglC is an OM lipoprotein, GltB and GltA are integral OM β-barrel proteins, and GltC is a soluble periplasmic protein. These findings are in agreement with the observation that the GltB and GltA paralogs NfsB and NfsA localize to the OM when expressed in *E*. *coli* [25]. The GltC paralog NfsC also fractionates with the OM in *E*. *coli* and has been suggested to associate with the OM [25]. Importantly, GltC and NfsC only share 19%/35% identity/similarity supporting the notion that these two proteins may interact differently with the OM.

### CglC faces the periplasmic side of the outer membrane

OM lipoproteins are commonly assumed to face towards the periplasm; however, several OM lipoproteins that are exposed on the cell surface have been identified [45–48]. To determine the orientation of CglC in the OM, intact WT *M*. *xanthus* cells were treated with limited amounts of Proteinase K. The OM β-barrel protein Oar [32,49,50] and the PilQ multimer in the OM were readily degraded (Fig 3B) while Tgl, which is an OM lipoprotein that faces the periplasm [51], and the IM protein PilC [43] showed little degradation in this assay. Importantly, CglC also only showed little degradation. Thus, we conclude that CglC is facing the periplasm.

### GltB and GltA are mutually stabilizing and stabilize GltC

**Fig 4:**
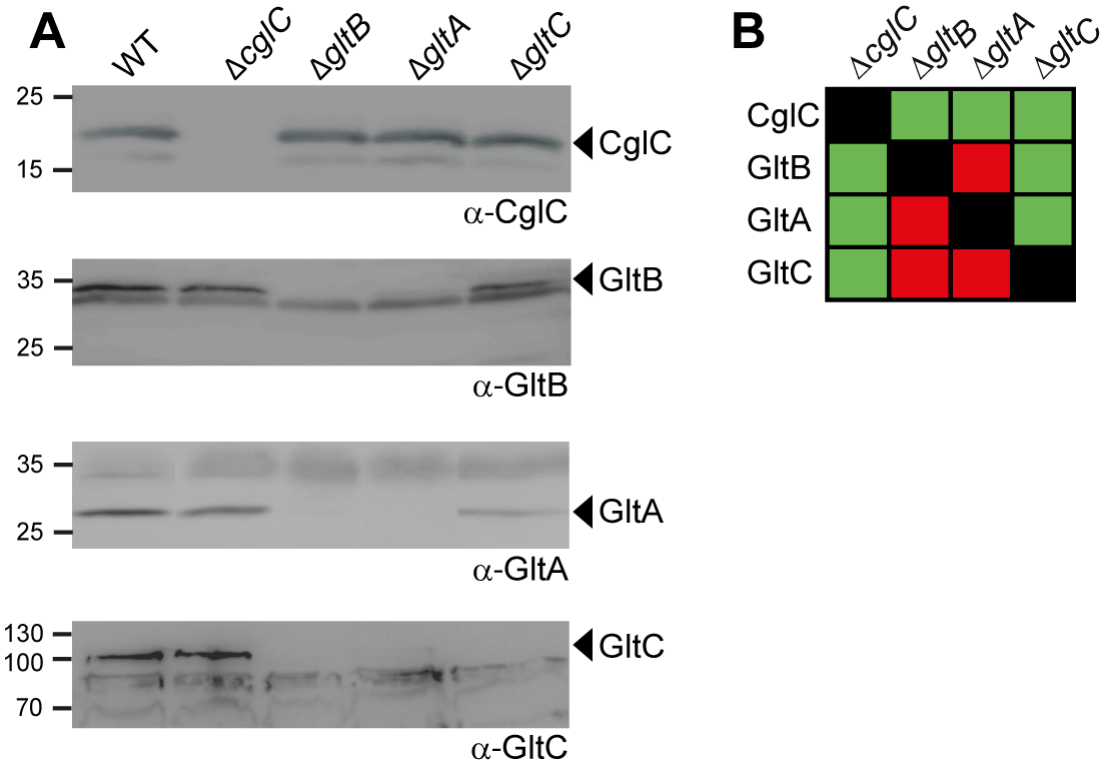
GltB and GltA are mutually stabilizing and stabilize GltC. (A) GltB and GltA are mutually stabilizing and stabilize GltC. Total cell extracts from cells grown as described in Fig 2D were isolated from strains of the indicated genotypes and analyzed by immunoblotting using specific antibodies as indicated. Molecular mass markers are indicated to the left. The relevant proteins are indicated. (B) Summary of observed effects on protein stability. Green and red indicate no effect or negative effect, respectively.

To analyze whether CglC, GltB, GltA or GltC may interact directly, we systematically determined the accumulation of each protein in the absence of each individual other protein using immunoblot analysis. CglC accumulated at WT levels in the absence of GltB, GltA or GltC and the absence of CglC did not affect the accumulation of GltB, GltA or GltC (Fig 4A and 4B). In the absence of GltB protein, GltA and GltC did not accumulate (Fig 4A and 4B). In the absence of GltA protein, GltB and GltC did not accumulate (Fig 4A and 4B). In the absence of GltC protein, GltB and GltA accumulated as in WT. These effects on protein accumulation were not caused by polar effects of the in-frame deletions because each of the in-frame deletion mutants was complemented by an ectopic copy of the relevant WT gene (Fig 2B and 2C and 2D). Additionally, the protein stability effects were not caused by the lack of gliding motility because GltB, GltA and GltC accumulated in the Δ*cglC* mutant. Similarly, GltB and GltA were stable in the absence of AglZ and AglQ (see below). We conclude that the two integral OM proteins GltB and GltA mutually stabilize each other as well as the periplasmic protein GltC suggesting that these three proteins interact directly. Supporting this conclusion, NfsA and NfsB are also mutually stabilizing and both are important for NfsC stability [24].

In the *M*. *xanthus* type IV pili system, the OM lipoprotein Tgl functions as a pilotin to stimulate multimer formation by the PilQ secretin in the OM [29]. To assess whether the OM lipoprotein CglC is important for the OM integration of GltB and GltA, we isolated the soluble fraction, the membrane fraction and OMVs from WT and Δ*cglC* cells and analyzed for the subcellular localization of GltB and GltA. In the absence of CglC protein, GltB and GltA accumulated in the membrane fraction (Fig 3C, left panel); however, the accumulation of GltB and GltA in the OMVs was significantly reduced (Fig 3C, right panel). Because GltB and GltA accumulate at WT levels in the Δ*cglC* mutant (Fig 4A and 4B), these data strongly suggest that CglC is important for integration of GltB and GltA into the OM and that GltB and GltA accumulate in the IM in the absence of CglC.

### GltB, GltA and GltC interact directly

To test for direct interactions, soluble tagged variants of CglC, GltB, GltA and GltC without their signal peptides were expressed and purified under native conditions from *E*. *coli*, i.e. MalE-CglC^20-172^, MalE-GltB^20-275^, MalE-GltA^22-256^, GltC^25-673^-His_6_ and GST-GltB^20-275^. Surprisingly, the OM β-barrel proteins GltB^20-275^ and GltA^22-256^ fused to MalE and also to GST in the case of GltB^20-275^ were soluble whereas C-terminally His_6_–tagged GltB^20-275^ and N-terminally His_6_-tagged GltA^20-256^ were not soluble suggesting that the MalE and GST tags help to maintain GltB^20-275^ and GltA^22-256^ solubility.

**Fig 5:**
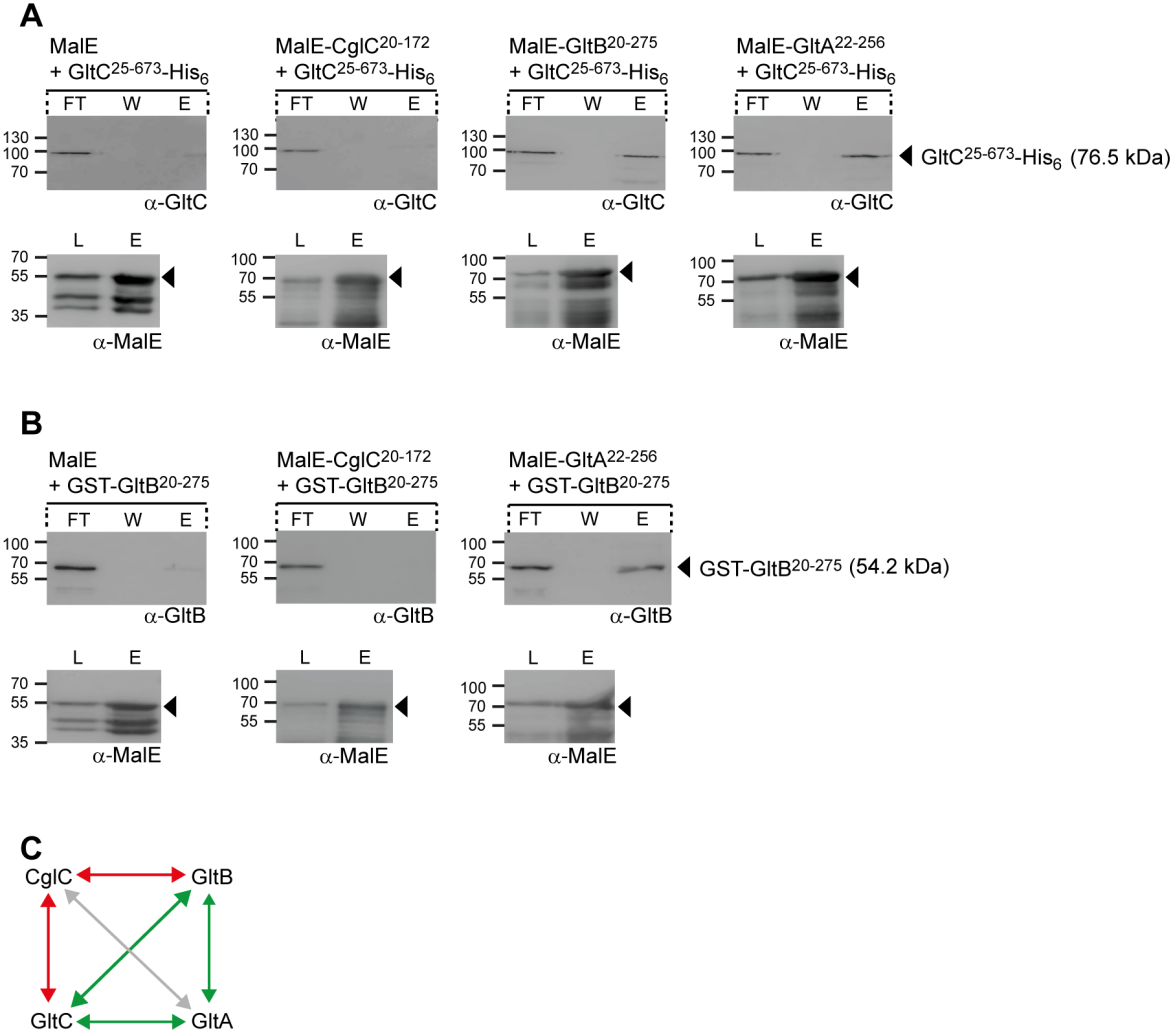
GltB, GltA and GltC interact directly. (A) GltC interacts with GltB and GltA. Purified MalE, MalE-CglC^20-172^, MalE-GltB^20-275^ or MalE-GltA^22-256^ were mixed with an equal amount of GltC^25-673^-His_6_, loaded on an amylose matrix, the matrix was washed and then bound proteins were eluted with 10 mM maltose. Samples before loading (L), from the flow through (FT), the washing step (W) and elution (E) were analyzed by immunoblotting using antibodies against GltC and MalE. In the upper row, arrowheads indicate GltC^25-673^-His_6_ with its calculated molecular mass and in the lower row, the individual MalE proteins. Molecular mass markers are indicated to the left. (B) GltB interacts with GltA. Purified MalE, MalE-CglC^20-172^ or MalE-GltA^22-256^ were mixed with an equal amount of GST-GltB^20-275^ and analyzed as described in A. Blots are marked as in A. (C) Summary of direct protein interactions. Green lines indicate interaction detected, red lines indicate interactions tested but not detected; grey line indicates interaction not tested.

To test for direct interactions, each MalE-tagged protein as well as MalE without an attached protein was incubated with an equal amount of GltC^25-673^-His_6_. Subsequently, the protein mixtures were loaded on an amylose-coupled matrix, bound proteins eluted by addition of maltose, separated by SDS-PAGE and analyzed by immunoblotting. In these experiments, GltC^25-673^-His_6_ specifically bound to the amylose matrix in the presence of MalE-GltB^20-275^ and MalE-GltA^22-256^ but not in the presence of MalE-CglC^20-172^ or MalE (Fig 5A and 5C).

In protein mixtures containing GST-GltB^20-275^ and MalE-CglC^20-172^, MalE-GltA^22-256^ or MalE, GST-GltB^20-275^ bound to the amylose matrix in the presence of MalE-GltA^22-256^ but not in the presence of MalE-CglC^20-172^ or MalE (Fig 5B). Therefore, GltB and GltA and GltC interact directly in all three pair-wise combinations whereas no interaction was observed between MalE-CglC^20-172^ and GltC^25-673^-His_6_ or GST-GltB^20-275^ (Fig 5C). These observations are in agreement with the *in vivo* protein stability experiments (Fig 4A and 4B). Because CglC^20-172^ and GltA^22-256^ could only be purified as soluble proteins when fused to MalE, we were unable to test for direct interactions between CglC and GltA. Our inability to detect an interaction between CglC and GltB despite the observation that CglC is important for OM integration of GltB and GltA indicate that such interactions may only be transient, that CglC interacts primarily with GltA, or this effect of CglC is indirect.

### GltB, GltA and GltC are components of the gliding motility complexes

To determine if CglC, GltB, GltA and GltC are components of the gliding motility complexes, we generated fluorescent proteins in which mCherry was fused to the C-terminus of the four proteins and then expressed under the control of the native promoter from plasmids integrated at the Mx8 *attB* site. The ectopic expression of the relevant fusion proteins restored the motility defects in the Δ*gltB*, Δ*gltA* and Δ*gltC* mutants and were not negative dominant in a WT background (S3A Fig), demonstrating that the fusion proteins are active. In the case of CglC-mCherry, we observed that the full-length fusion protein was degraded and therefore this construct was not considered further. GltB-mCherry (S3B Fig) and GltA-mCherry (S3C Fig) accumulated at levels comparable to those of the native proteins whereas GltC-mCherry accumulated at reduced levels (S3D Fig). Expression of GltB-mCherry in the Δ*gltB* background restored accumulation of GltA and GltC (S3F and S3G Fig). Similarly, expression of GltA-mCherry in the Δ*gltA* background restored accumulation of GltB and GltC (S3E and S3G Fig). As opposed to the native proteins (Fig 4A and 4B), GltB-mCherry accumulated at WT levels in the Δ*gltA* background (S3B Fig) and GltA-mCherry accumulated at WT levels in the Δ*gltB* background (S3C Fig) suggesting that the mCherry tag protects GltB and GltA from proteolytic degradation in the absence of GltA and GltB, respectively.

**Fig 6:**
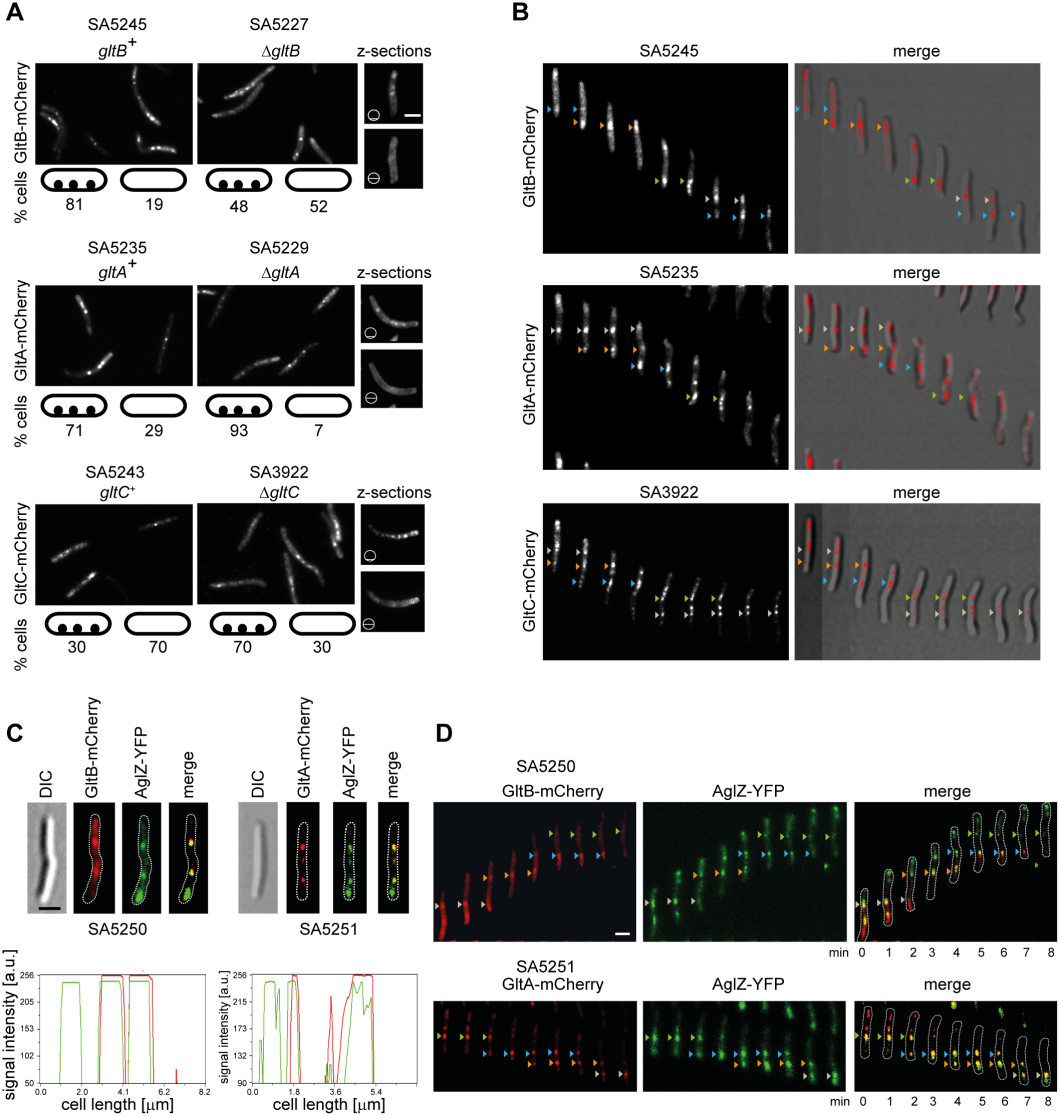
GltB and GltA are incorporated into gliding motility complexes. (A) Localization of GltB-mCherry, GltA-mCherry GltC-mCherry in the indicated genetic backgrounds. Cells were transferred from exponentially growing cultures to a thin agar pad on a microscope slide and imaged by fluorescence microscopy. For the z-sections, the z positions are indicated by a barred circle. The localization patterns observed are indicated in the schematics and numbers represent % of cells with that pattern; n > 100. Scale bar, 2μm. (B) Time-lapse microscopy of cells containing GltB-mCherry, GltA-mCherry or GltC-mCherry. Cells of the indicated genotypes were treated as in (A) and imaged by time-lapse DIC and fluorescence microscopy at 60 s intervals. Same colored arrowheads indicate position of motility complex during cell movement. Left panel, fluorescence microscopy images; right panel, merged DIC and fluorescence microscopy images. (C) Fluorescence microscopy images and line scans of cells expressing GltB-mCherry or GltA-mCherry and AglZ-YFP. In the line scans, red lines refer to GltB/GltA-mCherry while green lines refer to AglZ-YFP. Cells were treated as in A. Scale bar, 2μm. (D) Time-lapse microscopy of cells containing GltB-mCherry or GltA-mCherry and AglZ-YFP. Cells containing the indicated fusions were treated as in (A) and imaged by time-lapse fluorescence microscopy at 60 s intervals. Same colored triangles indicate position of motility complex during cell movement. The corresponding linescans are shown in S4 Fig.

In the WT background, GltB-mCherry localized to multiple clusters along the cell length in the majority of cells and in the Δ*gltB* background 48% of cells contained these clusters (Fig 6A) suggesting that GltB-mCherry is more efficiently incorporated into these clusters in the presence of native GltB. GltA-mCherry localized to multiple clusters along the cell length in the majority of cells in the WT as well as in the Δ*gltA* background (Fig 6A). GltC-mCherry also localized to multiple clusters along the cell length and did so more efficiently in the Δ*gltC* background (Fig 6A). In microscopy z-stacks of these cells, the GltB-mCherry, GltA-mCherry and GltC-mCherry clusters were only visible when the focal plane was focused close to the substratum (Fig 6A). In time-lapse fluorescence microscopy, all three proteins behaved similarly and formed fixed clusters that remained stationary with respect to the substratum as cells moved, assembled towards the leading cell pole, and disassembled towards the lagging cell pole (Fig 6B) suggesting that all three proteins are incorporated into the gliding motility complex.

To further establish that GltB, GltA and GltC are integral components of the gliding motility complex, we generated strains to localize GltB or GltA in parallel with AglZ, which localizes to the leading cell pole and to the clusters of gliding motility complexes [16]. As shown in Fig 6C and 6D and S4 Fig, GltB-mCherry as well as GltA-mCherry colocalized with AglZ-YFP to clusters along the cell length in both snapshot analyses and time-lapse analyses but did not colocalize with AglZ-AFP at the leading cell pole. From these analyses and because GltB, GltA and GltC interact directly, we conclude that GltB, GltA and GltC are components of the gliding motility complex.

### Incorporation of GltB and GltA into the gliding motility complex depends on proteins required for gliding motility

**Fig 7:**
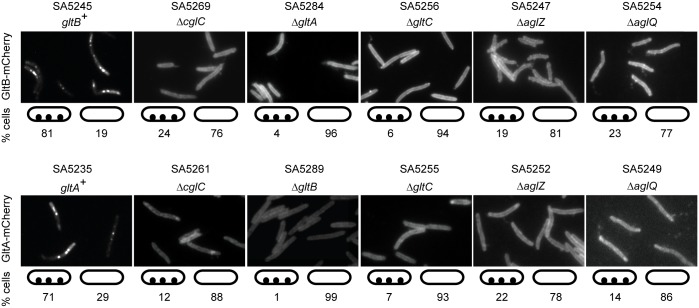
GltB and GltA incorporation into motility complexes depends on other gliding motility proteins. Cells were treated and analyzed as in Fig 6A. Note that all strains tested for GltB-mCherry and GltA-mCherry localization are *gltB* ^+^ and *gltA* ^+^, respectively. Scale bar, 2μm.

Next, we investigated the localization of GltB and GltA in the absence of other proteins required for gliding (Fig 7). Localization of GltB-mCherry to clusters along the cell length was strongly reduced in the absence of GltA, GltC, AglZ and AglQ and instead GltB-mCherry localized along the entire cell circumference suggesting that the fusion protein is homogeneously dispersed in the OM. GltA-mCherry localization to the clusters along the cell length was also dependent on GltB, GltC, AglZ and AglQ and in the absence of any one of these proteins GltA-mCherry also mostly localized along the entire cell circumference suggesting that the fusion protein is also homogeneously dispersed in the OM. GltB-mCherry and GltA-mCherry also mostly localized along the cell periphery in the absence of CglC, which our data suggest is important for OM integration of GltB and GltA, supporting the notion that in the absence of CglC, GltB and GltA are homogeneously dispersed in the IM. These observations confirm that GltB and GltA in the clusters along the cell length are incorporated into gliding motility complexes. Because native GltB and GltA (S5A Fig), as well as the GltB-mCherry and GltA-mCherry fusions (S5B Fig), accumulate as in WT in the absence of AglZ or AglQ, we conclude that incorporation of GltB and GltA, and by implication GltC, into the gliding motility complex depends on proteins that localize to the cytoplasm, IM, periplasm and OM.

### Incorporation of AglZ and AglQ into the gliding motility complex depends on CglC, GltB, GltA and GltC

**Fig 8:**
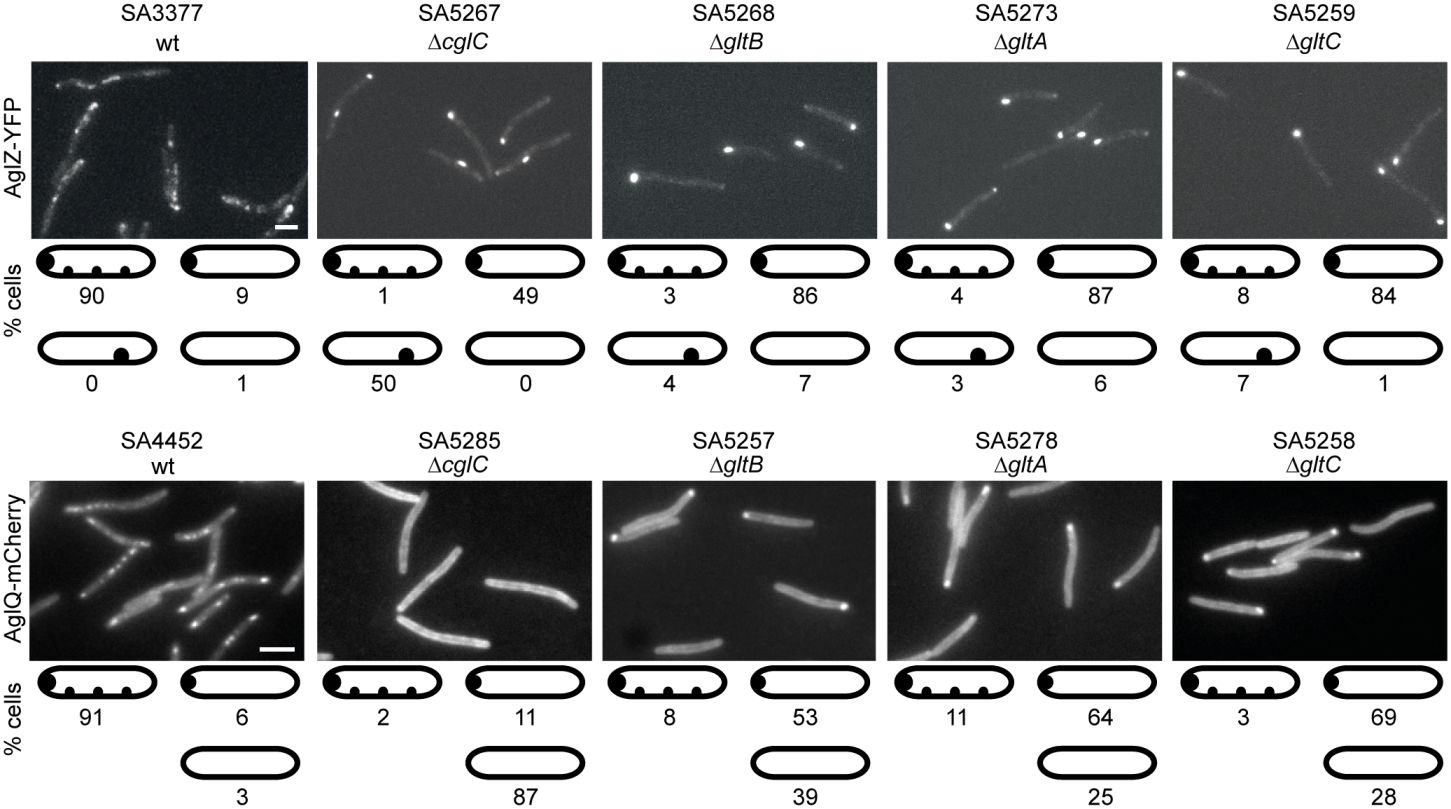
AglZ-YFP and AglQ-mCherry incorporation into motility complexes depends on CglC, GltB, GltA and GltC. Cells were treated and analyzed as in Fig 6A. Scale bar, 2μm.

To determine whether the incorporation of AglZ and AglQ into the gliding motility complex also depends on CglC, GltB, GltA and GltC, active AglZ-YFP and AglQ-mCherry fusions were expressed from their native sites in the four individual mutant backgrounds. As reported previously [16], AglZ-YFP localized to multiple clusters along the cell length in the majority of the cells as well as to a cell pole in the WT background (Fig 8). In contrast, in the four mutant backgrounds, AglZ-YFP predominantly formed a single large cluster that localized to a cell pole or somewhere along the cell length. These results were similar to those obtained with AglQ-mCherry, which also localize to multiple clusters along the cell length and a cell pole in the WT background [13] (Fig 8). In the absence of CglC, GltB, GltA or GltC, AglQ-mCherry mostly localized diffusely to the cell periphery as well as in a single cluster at a cell pole in the case of the Δ*gltB*, Δ*gltA* and Δ*gltC* mutants. In all four mutant backgrounds, the two fusion proteins accumulated as in WT (S6 Fig). We conclude that proteins that localize to the periplasm and OM are important for incorporation of AglZ as well as AglQ into the gliding motility complex.

### CglC stimulates assembly of the gliding motility complex after transfer

**Fig 9:**
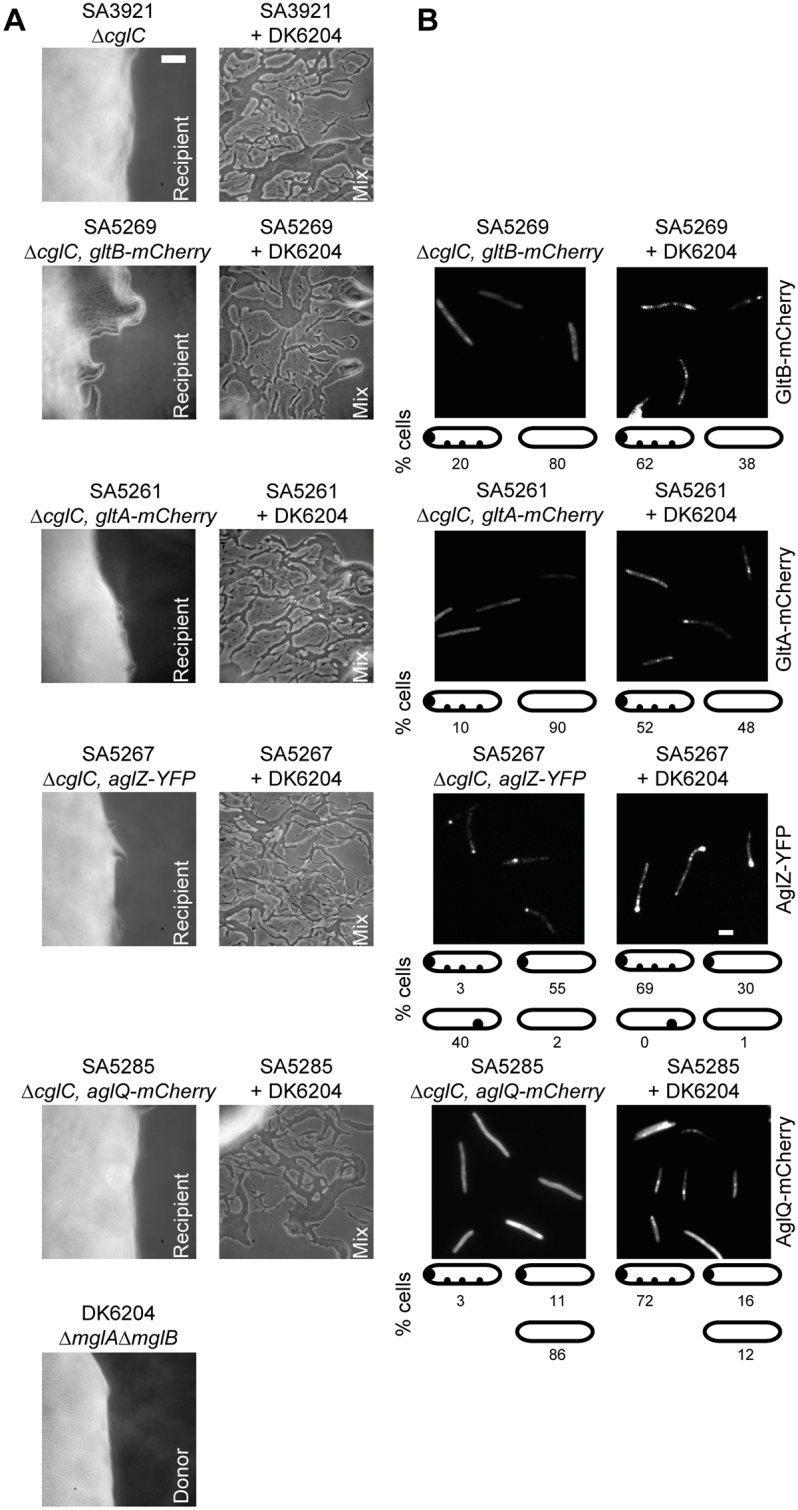
CglC stimulates formation of motility complexes after transfer. (A) Motility and stimulation phenotypes of the indicated strains and strain mixtures. Cells were incubated at 32°C for 48 h on 1.5% agar to score for gliding motility. Scale bar, 50μm. (B) Localization of GltB-mCherry, GltA-mCherry, AglZ-YFP and AglQ-mCherry in the Δ*cglC* mutant background incubated alone or in the presence of the non-motile CglC donor DK6204. Recipient and donor were mixed in a 1:1 ratio and incubated as described in (A), transferred to a thin agar pad on a microscope slide and imaged by fluorescence microscopy. Scale bar, 2μm.

Because CglC is important for the incorporation of GltB, GltA, AglZ, AglQ and likely also GltC into the gliding motility complexes, we hypothesized that transfer of CglC from a *cglC* ^+^ donor to a Δ*cglC* recipient instigates the assembly of intact motility complexes. To test this hypothesis, we performed stimulation assays in which the non-motile *cglC* ^+^ strain DK6204, which cannot be stimulated to move, served as a CglC donor as described [20] and five different Δ*cglC* strains served as recipients. DK6204 and the five Δ*cglC* strains had defects in gliding motility and had no single cells and slime trails at the colony edges (Fig 9A). Importantly, when the donor was mixed with a recipient, single cells and slime trails were readily observed at the colony edge, thus, verifying stimulation of the Δ*cglC* strains. As expected, GltB-mCherry, GltA-mCherry and AglQ-mCherry in the Δ*cglC* recipients mostly localized to the cell periphery in the absence of the donor (Fig 9B). Strikingly, all three proteins in the Δ*cglC* recipients in the presence of the donor strain localized in multiple clusters along the cell length in the majority of cells (Fig 9B). AglZ-YFP in the Δ*cglC* recipient in the absence of the donor mostly localized to a single cluster at a pole and also shifted towards formation of clusters along the cell length in the presence of the donor in the majority of cells (Fig 9B). Thus, during stimulation CglC from a donor brings about the assembly of intact and functional motility complexes in the recipient.

## Discussion

The machinery in *M*. *xanthus* that generates gliding motility has been hypothesized to span parts of the cell envelope or even the entire cell envelope. We report the functional characterization of the four proteins CglC, GltB, GltA and GltC all of which are required for gliding motility. We show that CglC is an OM lipoprotein that is facing towards the periplasm, GltB and GltA are integral OM β-barrel proteins, and GltC is a soluble periplasmic protein. The gliding motility machinery in *M*. *xanthus* is localized in clusters along the cell length. The localization of proteins required for gliding motility to such clusters is used as a readout for their incorporation into the motility machinery. We show that GltB, GltA and GltC localize to clusters along the cell length demonstrating that they are components of the gliding motility machinery. This conclusion is corroborated by several lines of evidence. First, GltB, GltA and GltC interact directly in all pair-wise combinations. Second, GltB and GltA colocalize with AglZ. Third, the localization of GltB and GltA to these clusters, in addition to being mutually dependent, was not only dependent on CglC and GltC but also on AglQ and AglZ that are *bona fide* components of the gliding motility machinery. Conversely, localization of AglZ and AglQ to these clusters, and therefore their incorporation into the gliding motility complex, was also dependent on CglC, GltB, GltA and GltC. In total, we conclude that GltB, GltA and GltC are integral components of the gliding motility machinery.

Previously identified components of the gliding motility machinery localize to the cytoplasm, IM and periplasm [13,15–18,23] (Fig 1). The identification of GltB and GltA as OM components of the gliding motility complex has implications for the understanding of the structure of this complex. The data presented are consistent with a focal adhesion model where the GltB/GltA/GltC subcomplex connects to proteins in the periplasm, IM and cytoplasm. Therefore, we assert that the gliding motility complex spans from the cytoplasm, over the IM and periplasm to the OM similarly to the machineries that drive flagella rotation and type IV pili extension/retraction. Notably, the motility complexes after assembly at the leading cell pole remain stationary with respect to the substratum as cells glide, therefore, this structural model of the gliding motility complex implies that these complexes “move through the peptidoglycan” [28]. None of the many proteins that are required for gliding motility contain peptidoglycan hydrolyzing or synthesizing domains. So, it remains an open question how the motility complexes “move through the peptidoglycan”. Similarly, it remains to be shown how the GltB/GltA/GltC subcomplex connect to the remaining proteins of the gliding machinery. However, several proteins including GltG and GltJ required for gliding motility have large periplasmic regions that could connect to the GltB/GltA/GltC subcomplex (Fig 1).

GltB and GltA mutually stabilize each other as well as GltC. Thus, if the GltB/GltA complex does not form, then these proteins and GltC become unstable. In the absence of AglZ or AglQ, GltB and GltA are not incorporated into the gliding motility complex, however, both proteins accumulate. The stability of GltB and GltA in the absence of AglZ or AglQ implies that GltB, GltA and GltC interact directly to form a subcomplex in the OM and periplasm under these conditions. In the absence of AglZ or AglQ, this subcomplex is dispersedly localized throughout the OM/periplasm and in the presence of AglQ and AglZ, this subcomplex is incorporated into the gliding motility complex. In the absence of GltB, GltA or GltC as well as in the absence of proteins predicted to localize to the cytoplasm, IM or periplasm [15,17] AglZ and AglQ mostly localize to a single cell pole suggesting that these two proteins engage in the formation of polarly localized subcomplexes in the absence of other components of the gliding machinery. Taken together these observations indicate that each component regardless of its cytoplasmic, IM, periplasmic or OM localization is crucial for the assembly of the gliding motility complex. This assembly may proceed via the connection of subcomplexes that are otherwise localized dispersedly to the OM/periplasm and IM/cytoplasm. This is in contrast to the assembly pathways of other motility complexes, where the assembly process essentially proceeds in a linear inside-out manner in the case of flagella systems [7] and in a linear outside-in manner in the case of type IV pili [38,52].

The role of CglC as an integral part of the gliding machinery remains unclear. In the case of the type IV pili machinery in *M*. *xanthus*, the OM lipoprotein Tgl functions as a pilotin and is important for assembly of the multimeric form of the PilQ secretin in the OM [29]. Because multimeric PilQ in the OM functions as an assembly platform for the remaining type IV pili machinery, lack of Tgl indirectly interferes with assembly of this entire machinery [38]. Similarly, the data presented here suggest that the sole function of CglC may be to facilitate the OM integration of GltB and GltA thereby allowing the assembly of the entire gliding motility machinery.

In 1977 Hodgkin and Kaiser observed that several gliding motility mutants including *cglC* mutants could be transiently stimulated to glide upon contact with a donor that was WT for the mutant gene [33] in a process that depends on the transfer of OM lipoproteins from the donor to the recipient [29]. A type II signal sequence that targets a protein to the OM is necessary and sufficient for transfer of that protein between cells [34,36]. The function of the transferred proteins and how this transfer results in stimulation of gliding has remained enigmatic. Here, we showed that in a Δ*cglC* mutant, the motility complexes do not assemble. However, upon stimulation, GltB, GltA, AglZ and AglQ localized to clusters along the cell length demonstrating that after stimulation, the gliding motility complexes assemble. Because our data suggest that CglC facilitates the OM incorporation of GltB and GltA, we propose that CglC provided from a donor to a recipient facilitates the OM incorporation of GltB and GltA in the recipient, thereby, allowing the remaining components of the machinery to assemble into functional gliding motility complexes. This is in striking analogy to the type IV pili system of *M*. *xanthus* in which the Tgl lipoprotein is transferred from a donor to a recipient and stimulates multimer formation of the PilQ secretin in the OM thereby allowing assembly of the type IV pili machinery [29,33,38].

The direct cell contacts required for OM lipoprotein transfer in *M*. *xanthus* depends on the cell surface receptor TraA [35,53]. TraA is highly polymorphic and transfer of OM lipoproteins depends on identical TraA proteins present in donor and recipient. Thus, the TraA polymorphism confers upon *M*. *xanthus* cells the ability to discriminate between self and non-self ensuring that OM lipoproteins are only shared with kin [53]. Recently, the molecular mechanisms underlying cell-cell contact-dependent activities in bacteria have started to be unraveled. The data presented here adds to this list by demonstrating that gliding motility can be stimulated in a contact-dependent manner by transfer of a protein that stimulates assembly of the gliding motility complexes.

## Materials and Methods

### Bacterial strains and cell growth

DK1622 was used as the WT *M*. *xanthus* strain and all *M*. *xanthus* strains used are derivatives of DK1622. *M*. *xanthus* strains used are listed in Table 1. Plasmids are listed in S1 Table. All plasmids were verified by sequencing. *M*. *xanthus* cells were grown at 32°C in 1% CTT broth [33] and on CTT agar plates supplemented with 1.5% agar. Kanamycin (50 μg/ml) or oxytetracycline (10 μg/ml) was added when appropriate. Plasmids were introduced into *M*. *xanthus* by electroporation. Site-specific integration of plasmids at the Mx8 *attB* site on the chromosome was confirmed by PCR. In frame deletions and gene replacements were generated as described [54,55].

**Table 1:** *M*. *xanthus* strains used in this work^1^.

Strain	Relevant characteristics	Reference
DK1622	Wild type	[70]
DK1217	*aglB*	[71]
DK1300	*sglG1*	[71]
DK8615	Δ*pilQ*	[72]
DK10417	Δ*pilC*	[73]
DK10405	Δ*tgl*::*Tc*	[39]
SA2273	P*_nat_-romR-GFP* (pSH1208)	[62]
MxH2265	Δ*aglZ*	[74]
SA3377	*aglZ-yfp* (pSL65*)	[75]
SA5293	Δ*aglQ*	This work
SA5309	P*_nat_-romR-GFP* (pSH1208), MXAN_2541::miniHimarΩ3908	This work
SA5347	P*_nat_-romR-GFP* (pSH1208), MXAN_2540::miniHimarΩ3927	This work
SA3921	Δ*cglC*	This work
SA3922	Δ*gltB*	This work
SA3923	Δ*gltA*	This work
SA3924	Δ*gltC*	This work
SA5544	Δ*oar*	This work
SA3935	Δ*cglC*, P*_pilA_-cglC* (pDK110)	This work
SA5911	Δ*gltB*, P*_pilA_-gltB* (pDK111)	This work
SA5912	Δ*gltA*, P*_pilA_-gltA* (pDK112)	This work
SA3938	Δ*gltC*, P*_pilA_-gltC* (pDK113)	This work
SA4452	*aglQ-mCherry*	This work
SA5209	Δ*MXAN_2542*	This work
SA5242	Δ*cglC*, *P_nat_-cglC* (pBJA31)	This work
SA5239	Δ*gltB*, P*_nat_-gltB* (pBJA32)	This work
SA5228	Δ*gltA*, P*_nat_-gltA* (pBJA33)	This work
SA5246	Δ*gltC, P_nat_-gltC* (pBJA34)	This work
SA5245	P*_nat_-gltB-mCherry* (pBJA35)	This work
SA5269	Δ*cglC*, P*_nat_-gltB-mCherry* (pBJA35)	This work
SA5227	Δ*gltB*, P*_nat_-gltB-mCherry* (pBJA35)	This work
SA5284	Δ*gltA*, P*_nat_-gltB-mCherry* (pBJA35)	This work
SA5256	Δ*gltC*, P*_nat_-gltB-mCherry* (pBJA35)	This work
SA5235	P*_nat_-gltA-mCherry* (pBJA36)	This work
SA5261	Δ*cglC*, P*_nat_-gltA-mCherry* (pBJA36)	This work
SA5289	Δ*gltB*, P*_nat_-gltA-mCherry* (pBJA36)	This work
SA5229	Δ*gltA*, P*_nat_-gltA-mCherry* (pBJA36)	This work
SA5255	Δ*gltC*, P*_nat_-gltA-mCherry* (pBJA36)	This work
SA5247	Δ*aglZ*, P*_nat_-gltB-mCherry* (pBJA35)	This work
SA5254	Δ*aglQ*, P*_nat_-gltB-mCherry* (pBJA35)	This work
SA5252	Δ*aglZ*, P*_nat_-gltA-mCherry* (pBJA36)	This work
SA5249	Δ*aglQ*, P*_nat_-gltA-mCherry* (pBJA36)	This work
SA5243	P*_nat_-gltC-mCherry* (pBJA37)	This work
SA5244	Δ*gltC*, P*_nat_-gltC-mCherry* (pBJA37)	This work
SA5267	Δ*cglC*, *aglZ-yfp* (pSL65*)	This work
SA5268	Δ*gltB*, *aglZ-yfp* (pSL65*)	This work
SA5273	Δ*gltA*, *aglZ-yfp* (pSL65*)	This work
SA5259	Δ*gltC*, *aglZ-yfp* (pSL65*)	This work
SA5285	Δ*cglC*, *aglQ-mCherry*	This work
SA5257	Δ*gltB*, *aglQ-mCherry*	This work
SA5278	Δ*gltA*, *aglQ-mCherry*	This work
SA5258	Δ*gltC*,*aglQ-mCherry*	This work
SA5250	P*_nat_-gltB-mCherry*, *aglZ-yfp* (pBJA35, pSL65*)	This work
SA5251	P*_nat_-gltA-mCherry*, *aglZ-yfp* (pBJA36, pSL65*)	This work

^1^ Plasmids mentioned in parentheses were integrated at the chromosomal Mx8 *attB* site with the exception of those marked with an asterisk (*), which were integrated at the native site by a single homologous recombination event. Constructs were transcribed from the native promoter (P*nat*) or the *pilA* promoter (P*pilA*) as indicated.

### Transposon mutagenesis

The transposon miniHimar(Kan) on the plasmid pMiniHimar, which is a non-replicating plasmid in *M*. *xanthus* (X. Duan and H.B. Kaplan, personal communication), was introduced into the strain SA2273 by electroporation. Transformants were selected on the basis of their resistance to kanamycin and individually transferred to plates with 0.5% CTT supplemented with 1.5% agar and 50 μg kanamycin/ml and incubated at 32°C to test for gliding motility defects. After 24 hrs, mutant strains were scored for motility defects. A total of 15,000 transformants were isolated and screened for gliding motility defects. Among these, 36 were deficient in gliding motility. The transposon insertion sites were identified by arbitrary PCR with subsequent sequencing as described [56,57]. Sequences were examined against the *M*. *xanthus* genome using BLASTn [58] to identify insertion sites. The entire collection of mutants will be described elsewhere.

### Motility assays

Cells from exponentially growing cultures were concentrated to density of 5 × 10^9^ cells/ml in TPM buffer (10 mM Tris-HC1 pH 7.5, 1 mM KH_2_PO_4_, 8 mM MgSO_4_). 5 μl of the cell suspensions were spotted on 0.5% CTT plates containing 0.5% agar or 1.5% agar [42] and incubated at 32°C for 24 h. Colony edges were visualized with a Leica MZ75 stereomicroscope equipped with a Leica DFC280 camera or a Leica DM6000B microscope equipped with a Cascade II camera.

### Operon mapping

Total RNA was isolated from exponentially growing *M*. *xanthus* DK1622 cells in 1.0% CTT using a hot phenol extraction method [59]. Subsequently, RNA was treated with DNase I (Ambion) and purified with the RNeasy kit (Qiagen). cDNA was synthesized from 1.0 μg total RNA using the High capacity cDNA Archive kit (Applied Biosystems) and random hexamer primers. Total RNA and genomic DNA was used as negative and positive controls, respectively. SYBR Green PCR Master Mix (Applied Biosystems) was added to cDNA from the reverse transcription of 30 ng RNA together with 100 nM each of the two primers. The RT-PCR reaction was performed on a Mastercycler personal (Eppendorf).

### Stimulation assay

Cells were treated as described for motility assay. Donor and recipient cell suspensions were spotted separately as well as in a 1:1 mixture on 0.5% CTT plates containing 1.5% agar and incubated at 32°C for 48 h. Colony edges were imaged as described for motility assays after 48 hrs of incubation. To visualize AglZ-YFP and AglQ-mCherry, cells from the edges of the colonies were harvested and resuspended in 30 μl CTT, spotted on 1.5% agar pads supplemented with TPM, covered with a cover slip and imaged as described for fluorescence microscopy.

### Fractionation experiments

Cells were fractionated as described [60]. Briefly, cells were grown exponentially to a density of 5 × 10^8^ cells/ml. Cells were harvested by centrifugation at 8000×g at RT for 10 min and resuspended in 50 mM Tris-HCl pH 7.6 containing “complete Protease inhibitor cocktail” (Roche). Cells were disrupted by sonication and samples were centrifuged at 3000×g at 4°C for 10 min to remove cell debris. Subsequently, the supernatant was centrifuged at 45.000×g at 4°C for 30 min to separate soluble and insoluble (membrane-associated) components. The resulting supernatant is enriched in soluble cytoplasmic and periplasmic proteins. The pellet containing a crude envelope fraction was washed with 50 mM Tris-HCl, resuspended in 50 mM Tris-HCl pH 7.2, 2% Triton X-100, 10 mM MgCl_2_ and incubated overnight with gentle shaking at 4°C and then subjected to centrifugation at 45.000×*g* for 30 min at 4°C. The resulting supernatant is enriched in membrane proteins. OMVs were isolated as described [32]. Briefly, culture supernatants were passed through a 0.2μm vacuum filter (Millipore). The resulting filtrate was centrifuged at 150000×g for 2 h at 4°C to recover membrane vesicles. The supernatant was carefully removed and the vesicle pellet was resuspended in 50 mM Tris-HCl pH 8.0 and centrifuged at 150000×g for 2 h at 4°C to concentrate vesicles.

### Protease accessibility experiments

1 ml of exponentially growing *M*. *xanthus* cells in CTT were directly incubated with Proteinase K (Sigma) with gentle shaking at 32°C for 10 min at the indicated concentrations. 100 μl of solution 1 (1 tablet “complete mini-protease inhibitor cocktail” (Roche) dissolved in 1 ml CTT) was added to stop the reaction. The cell suspension was centrifuged at 16.000×g at 4°C for 5 min. The cell pellet was washed with 1 ml of solution 2 (1 tablet “complete mini-protease inhibitor cocktail” dissolved in 10ml CTT) and resuspended in solution 1 to a calculated density of 1×10^10^ cells/ml. Samples were incubated at 100°C for 15 min and mixed with SDS loading buffer containing protease inhibitors (1 tablet “Complete mini-protease inhibitor cocktail” dissolved in 1ml SDS lysis buffer) to a density of 5×10^9^ cells/ml.

### Immunoblotting

Cells from exponentially growing cultures were concentrated and resuspended in SDS lysis buffer to a density of 2.5 × 10^9^ cells/ml. Proteins from the same number of cells were loaded per lane. Immunoblots were performed using standard procedures [61] with polyclonal rabbit α-CglC, α-GltB, α-GltA, α-GltC, α-PilB [44], α-PilC [43], α-PilQ [43], α-Oar, α-MalE (New England Biolabs), α-mCherry (Roche) and secondary anti-rabbit immunoglobulin G peroxidase conjugate (Sigma). For YFP-tagged protein detection, monoclonal anti-GFP mouse antibodies (Roche) and peroxidase-conjugated rabbit anti-mouse immunoglobulin G secondary antibodies (DakoCytomation) were used. Immunoblots were developed using Luminata Western HRP Substrate (Merck Millipore). The α-CglC, α-GltB, α-GltA, α-GltC antibodies were generated against purified His_6_-tagged proteins (see below). α-Oar antibodies were generated against the Oar peptides AB1 (^237^GTLEGTRKGIREEGT) and AB2 (^1009^SVDGDVNKNFKNPLS). Oar has a length of 1037 amino acid residues without the signal peptide.

### Protein purification

To purify GltC^25-673^-His_6_, MalE-CglC^20-172^, MalE-GltB^20-275^, MalE-GltA^22-256^ and GST-GltB^20-275^ the plasmids pBJA9, pBJA26, pBJA27, pBJA28, pBJA29 were introduced into *E*. *coli* Rosetta 2 [F-*ompT hsdSB*(rB-mB-) *gal dcm* pRARE2] (Novagen). Cultures were grown in LB medium at 37°C to OD_600_ = 0.7. Protein expression was induced by addition of IPTG to 0.1mM final concentration. Proteins were expressed overnight at 18°C. Cells were harvested by centrifugation at 9.000×g for 10 min at RT and resuspended in lysis buffer containing “EDTA-free complete protease inhibitor cocktail” (Roche). For MalE- and GST-tagged proteins, CB1 lysis buffer (20 mM Tris-HCl pH7.4, 200 mM NaCl, 1 mM DTT, 1 mM EDTA) was used while for His_6_-tagged proteins the lysis buffer was (50 mM NaH_2_PO_4_, 300 mM NaCl pH 7.5, 1 mM DTT). Harvested cells were disrupted by sonication and centrifuged at 20.500×g for 30 min at 4°C to remove debris.

MalE-tagged proteins were purified on amylose matrix column (New England Biolabs) and eluted with CB1 buffer supplemented with 10 mM maltose. GltC^25-673^-His_6_ was purified on Ni^2+^-NTA-agarose column as recommended by the manufacturer (Qiagen) and eluted with elution buffer (50 mM NaH_2_PO_4_, 300 mM NaCl, 250 mM imidazole, pH 8.0). GST-GltB^20-275^ was purified on GST-bind column (Novagen) and eluted with CB1 buffer supplemented with 10 mM reduced glutathione.

His_6_-CglC^20-172^, GltB^20-275^-His_6_ and His_6_-GltA^22-256^ were purified from inclusion bodies. The plasmids pBJA1, pBJA10, pBJA3 were introduced into *E*. *coli* Rosetta 2. Cultures were grown as described. Cells were resuspended in lysis buffer (50 mM NaH_2_PO_4_, 300 mM NaCl pH 7.5, 1 mM DTT) containing “EDTA-free complete Protease inhibitor cocktail” (Roche), sonicated and harvested as described. The cell pellet was resuspended in buffer B (100 mM NaH_2_PO_4_, 10 mM TrisHCl, 8 M urea, pH 8.0) and incubated overnight at 4°C. The mixture was centrifuged at 20.500×g for 30 min at 4°C to remove cell debris. Proteins were purified on Ni^2+^-NTA-agarose column as described by the manufacturer (Qiagen). The column was washed with buffer C (buffer B; pH 6.3) and proteins were eluted with buffer D (buffer B, pH 5.9) and buffer E (buffer B, pH 4.5). The purified proteins were dialyzed against buffer B containing 10% glycerol, frozen in liquid nitrogen, stored at -80°C and used for polyclonal rabbit antibody generation.

### Pull-down experiments

Purified proteins were dialyzed against CB1 buffer. Protein concentration was measured by using a Bradford assay (BioRad). In pull-down experiments, 300 μg of each protein were mixed in a total volume of 1 ml of buffer CB1 and incubated at 4°C for 1 h. Subsequently, these mixtures were incubated with an amylose matrix (New England Biolabs) for 2 h at 4°C, followed by a washing step with 10-times the matrix volume of buffer CB1. Proteins were eluted with buffer CB1 supplemented with 10 mM maltose.

### Fluorescence microscopy

DIC and fluorescence microscopy was performed as described [62]. Briefly, cells from exponentially growing cultures were spotted on 1.5% agar pads supplemented with TPM or A50 (10 mM MOPS pH 7.2, 1 mM CaCl_2_, 1 mM MgCl_2_, 50 mM NaCl, 1.5% agar) and covered with a cover slip. Cells were incubated at RT for 15 min to attach to the agar surface. Microscopy was performed using a Leica DMI6000B microscope with an adaptive focus control, a motorized stage, a temperature-controlled stage and a Hamamatsu Flash 4.0 camera. Images were recorded with Leica MM AF software package and processed with Metamorph (Molecular Devices).

### Sequence analysis of gliding machinery components

We performed sequence analysis of all of the gliding machinery components included in Fig 1. Type I and type II signal peptides were identified using the SignalP [63] and LipoP [64] webservers, respectively. Trans-membrane α-helices were identified using the DAS webserver [65]. OM β-barrel domains were identified using HHomp [66]. The HMMER3 software package [67] was used in conjunction with the Pfam26 domain library [68] for domain architecture analysis with default gathering thresholds. In the event of domain overlaps, the highest scoring domain model was chosen for the final domain architecture.

## Supporting Information

S1 Fig *gltB*, *gltA* and *gltC* are co-transcribed. DNA fragments labeled 1, 3, 5 or 7 cover intergenic regions while DNA fragments labeled 2, 4, 6 or 8 are fragments internal to genes. Numbers indicated above each lane correspond to that particular DNA fragment. Genomic DNA, total RNA and cDNA were used as templates in the indicated reactions.
(TIF)

S2 Fig Predicted topology of the OM β-barrel proteins GltB and GltA. The primary amino acid sequences of GltB and GltA without their signal peptide was analyzed using the software BOCTOPUS [69] to predict the topology of the β-strands in the β-barrel. Trans-membrane β-strands are indicated by black arrows overlapping the OM (grey). Note that the N-terminal 80 residues of GltB and 45 residues of GltA are not included.
(TIF)

S3 Fig Analysis of GltB-mCherry, GltA-mCherry and GltC-mCherry fusion proteins. (A) Gliding motility assay on 1.5% agar. Images of the colony edges were taken after 24 h incubation at 32°C. Scale bar = 50μm. (B-G) Immunoblot analysis of *M*. *xanthus* strains expressing GltB-mCherry, GltA-mCherry or GltC-mCherry under the control of native promoter at the Mx8 *attB* site. Bands corresponding to the fusion proteins are marked with grey triangles while native proteins are marked with black triangles.
(TIF)

S4 Fig GltB and GltA colocalize with AglZ. Cells containing the indicated fusions were treated as in Fig 6 and imaged by time-lapse fluorescence microscopy at 60 s intervals. Same colored triangles indicate position of focal adhesion during cell movement. In the line scans, red lines refer to GltB/GltA-mCherry while green lines refer to AglZ-YFP. The upper set of line scans represent the GltB/AglZ strain and the lower set of line scans represent the GltA/AglZ strain. Note that the fluorescence images are identical to those shown in Fig 6D.
(TIF)

S5 Fig GltB and GltA accumulate independently of AglZ and AglQ. (A, B) Immunoblot analysis of the accumulation of GltB and GltA in Δ*aglZ* and Δ*aglQ* strains (A) and of GltB-mCherry or GltA-mCherry under the control of native promoter at the Mx8 *attB* site in Δ*aglZ* and Δ*aglQ* strains (B). Bands corresponding to the native proteins and fusion proteins are marked with black and grey triangles, respectively together with their calculated molecular masses.
(TIF)

S6 Fig AglZ-YFP and AglQ-mCherry accumulate independently of CglC, GltB, GltA and GltC. Immunoblot with GFP—or mCherry primary antibodies. Bands corresponding to the fusion proteins are marked with the arrow heads together with the calculated molecular mass of the fusion proteins.
(TIF)

S1 Table Plasmids used in this work. (DOC)
