# Supplementary figures and images for: Contact- and Protein Transfer-Dependent Stimulation of Assembly of the Gliding Motility Machinery in Myxococcus xanthus
Source: PLoS Genet. 2015 Jul 1;11(7):e1005341. doi: 10.1371/journal.pgen.1005341 (PMC4488436; doi:10.1371/journal.pgen.1005341)

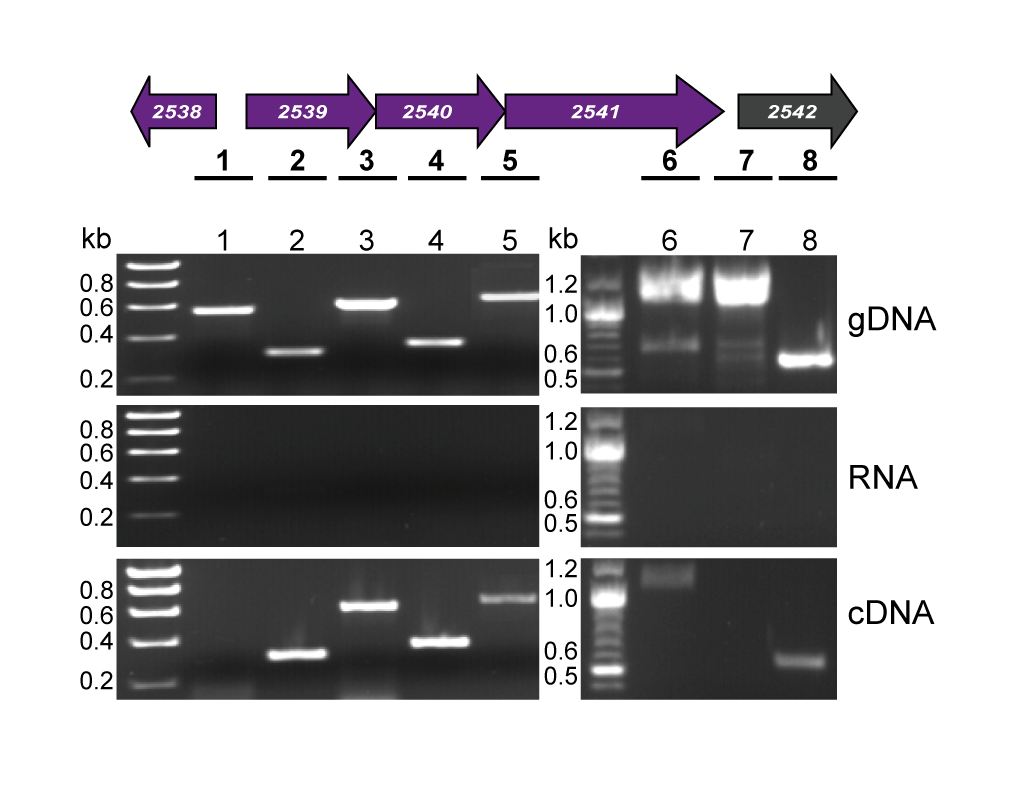

Supplement: S1 Fig — DNA fragments labeled 1, 3, 5 or 7 cover intergenic regions while DNA fragments labeled 2, 4, 6 or 8 are fragments internal to genes. Numbers indicated above each lane correspond to that particular DNA fragment. Genomic DNA, total RNA and cDNA were used as templates in the indicated reactions. (TIF) [file pgen.1005341.s001.tif]

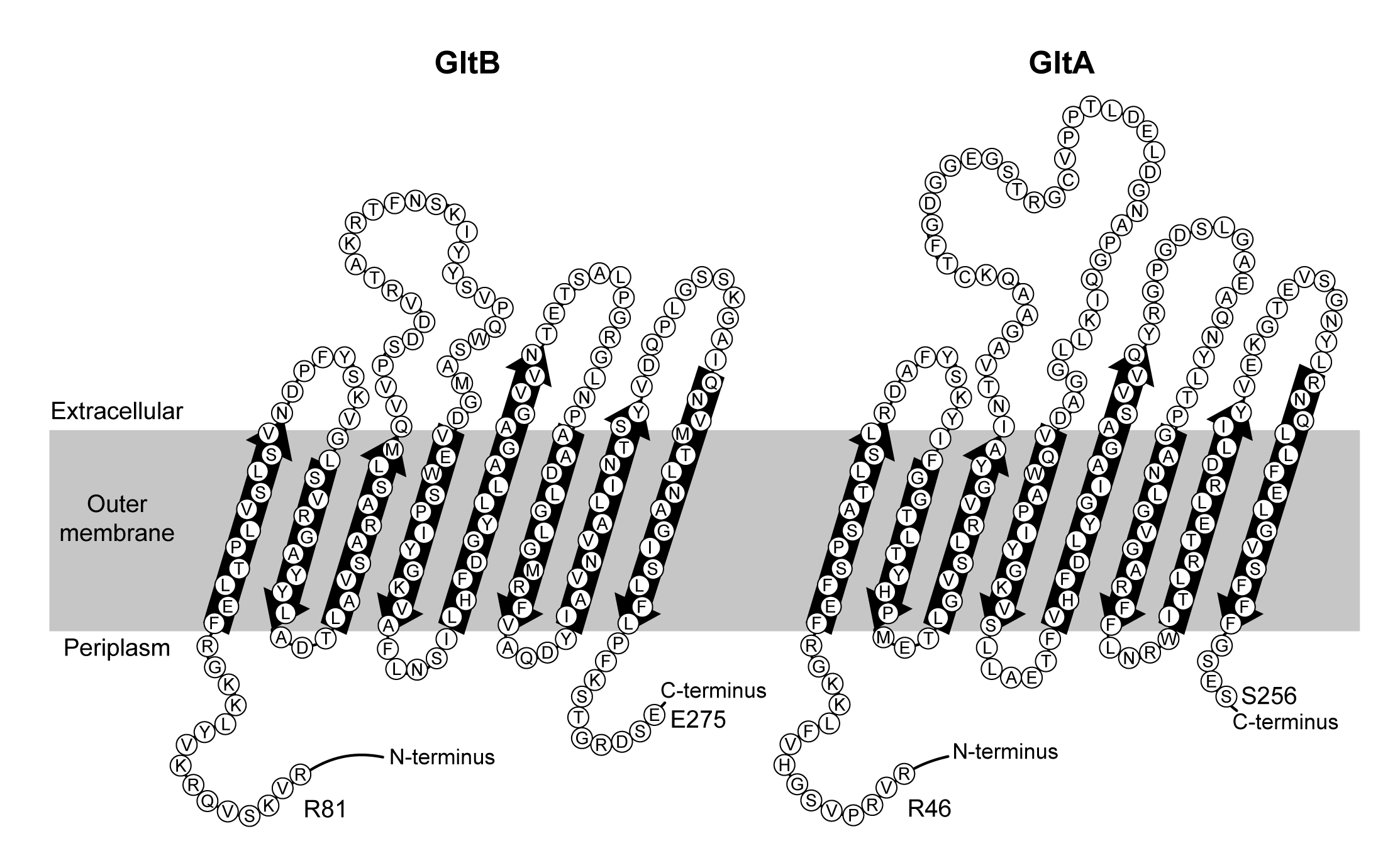

Supplement: S2 Fig — The primary amino acid sequences of GltB and GltA without their signal peptide was analyzed using the software BOCTOPUS [69] to predict the topology of the β-strands in the β-barrel. Trans-membrane β-strands are indicated by black arrows overlapping the OM (grey). Note that the N-terminal 80 residues of GltB and 45 residues of GltA are not included. (TIF) [file pgen.1005341.s002.tif]

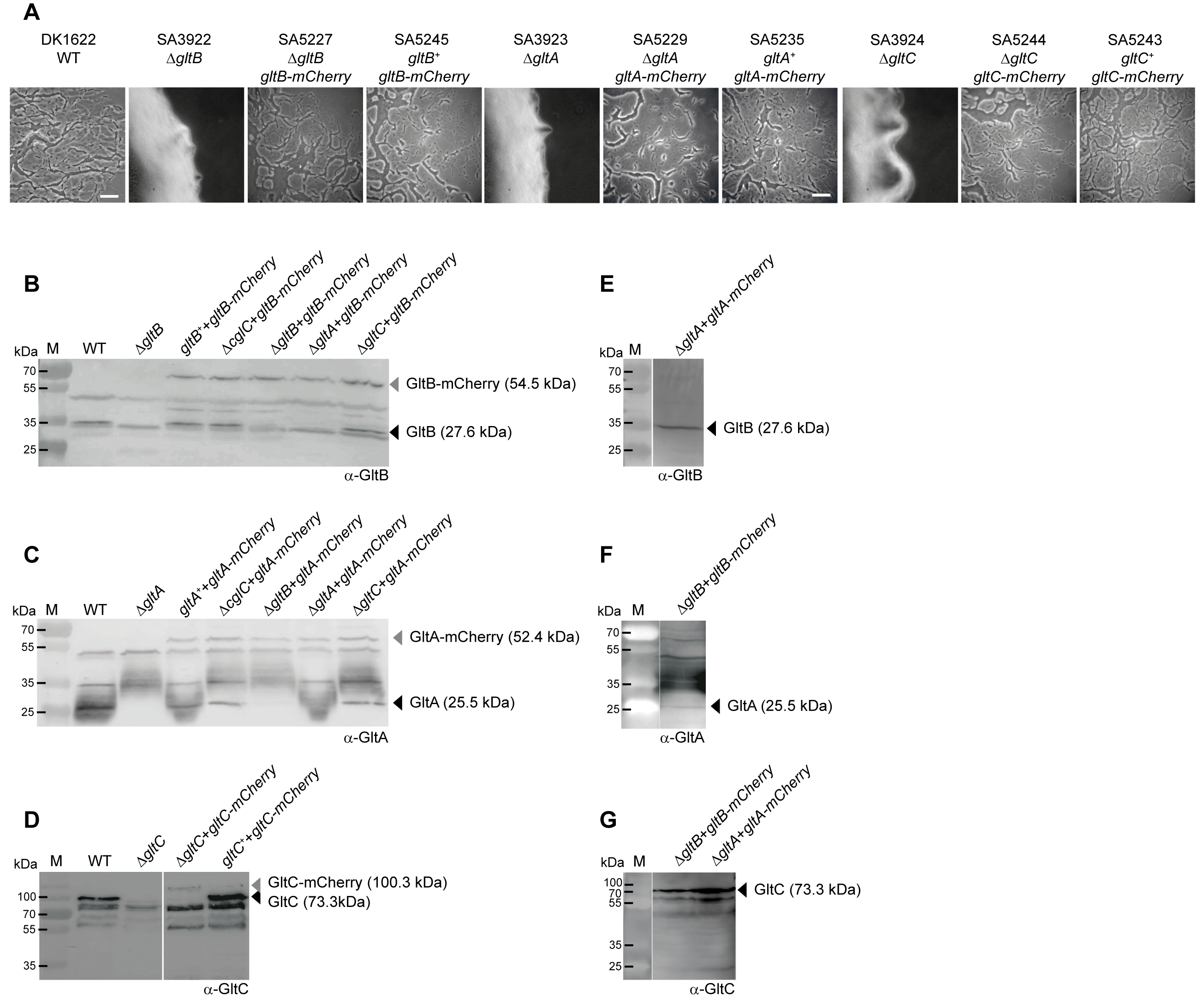

Supplement: S3 Fig — (A) Gliding motility assay on 1.5% agar. Images of the colony edges were taken after 24 h incubation at 32°C. Scale bar = 50μm. (B-G) Immunoblot analysis of M. xanthus strains expressing GltB-mCherry, GltA-mCherry or GltC-mCherry under the control of native promoter at the Mx8 attB site. Bands corresponding to the fusion proteins are marked with grey triangles while native proteins are marked with black triangles. (TIF) [file pgen.1005341.s003.tif]

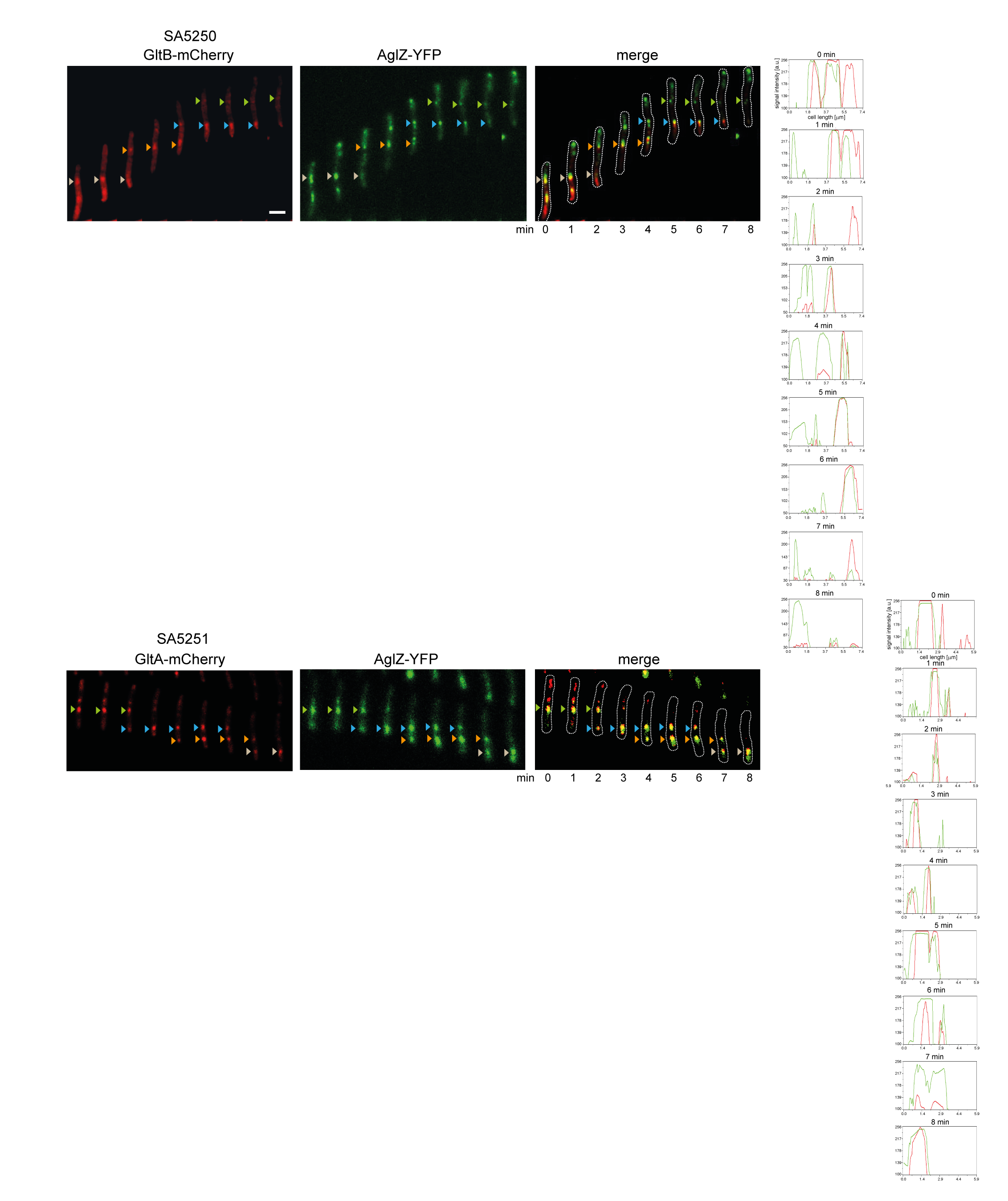

Supplement: S4 Fig — Cells containing the indicated fusions were treated as in Fig 6 and imaged by time-lapse fluorescence microscopy at 60 s intervals. Same colored triangles indicate position of focal adhesion during cell movement. In the line scans, red lines refer to GltB/GltA-mCherry while green lines refer to AglZ-YFP. The upper set of line scans represent the GltB/AglZ strain and the lower set of line scans represent the GltA/AglZ strain. Note that the fluorescence images are identical to those shown in Fig 6D. (TIF) [file pgen.1005341.s004.tif]

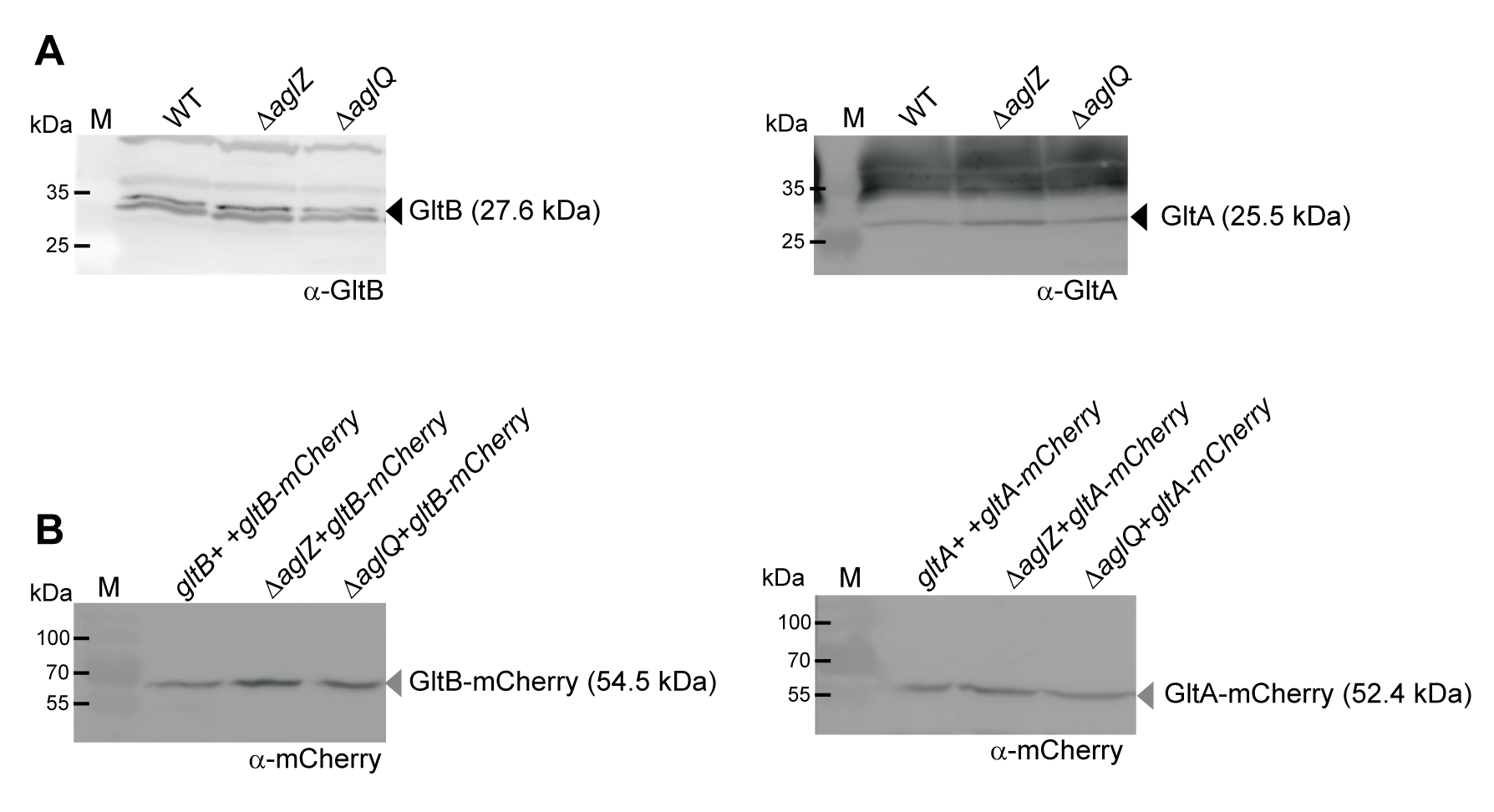

Supplement: S5 Fig — (A, B) Immunoblot analysis of the accumulation of GltB and GltA in ΔaglZ and ΔaglQ strains (A) and of GltB-mCherry or GltA-mCherry under the control of native promoter at the Mx8 attB site in ΔaglZ and ΔaglQ strains (B). Bands corresponding to the native proteins and fusion proteins are marked with black and grey triangles, respectively together with their calculated molecular masses. (TIF) [file pgen.1005341.s005.tif]

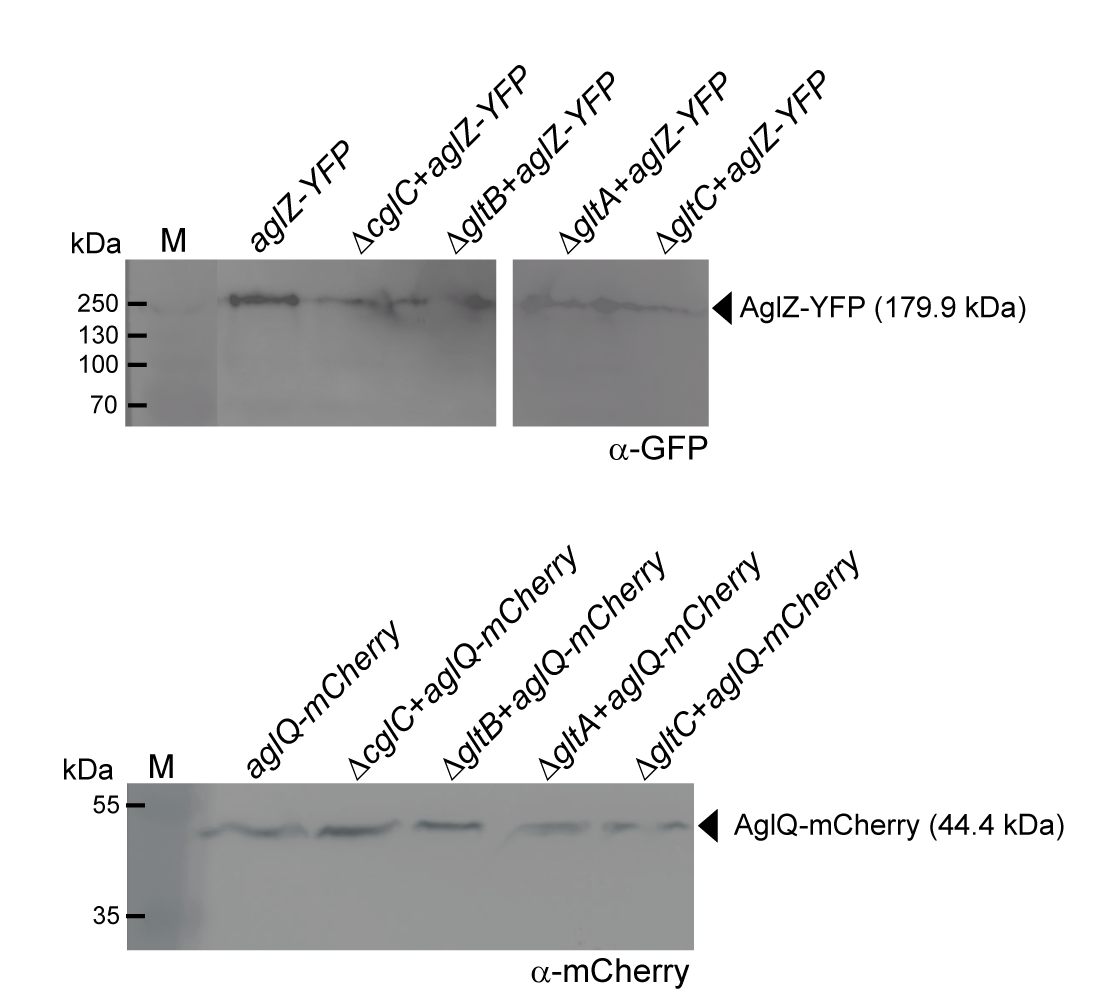

Supplement: S6 Fig — Immunoblot with GFP—or mCherry primary antibodies. Bands corresponding to the fusion proteins are marked with the arrow heads together with the calculated molecular mass of the fusion proteins. (TIF) [file pgen.1005341.s006.tif]
