# Supplementary material for: Contact- and Protein Transfer-Dependent Stimulation of Assembly of the Gliding Motility Machinery in Myxococcus xanthus
Source: PLoS Genet. 2015 Jul 1;11(7):e1005341. doi: 10.1371/journal.pgen.1005341 (PMC4488436; doi:10.1371/journal.pgen.1005341)
Supplement: S1 Table — (DOC) [file pgen.1005341.s007.doc]

**Table S1. Plasmids used in this work**

| **Name** | **Description** | **Source or reference** |
| --- | --- | --- |
| **pGEX-4T1** | Vector for GST overexpression | GE-Healthcare |
| **pCHYC** | Vector for *mCherry* amplification | M. Thanbichler |
| **pBlueskript II** | cloning vector | Fermentas |
| **pET24 b^+^** | Allowing generation and overexpression of C-terminal His_6_-tagged fusions | Merck Millipore |
| **pET45b^+^** | Allowing generation and overexpression of N-terminal His_6_-tagged fusions | Merck Millipore |
| **pMal-c2x** | Vector for MalE overexpression | New England biolabs |
| **pBJ114** | Vector for generation of in-frame deletions |  |
| **pSWU30** | Allowing gene expression from a chosen promoter after integration at the Mx8 *attB* site | D. Kaiser |
| **pSW105** | Allowing gene expression from the P*_pilA_* promoter after integrations at the Mx8 *attB* site |  |
| **pSL65** | Allowing insertion of pBJ113 *aglZ-yfp* at the *aglZ* native site |  |
| **pBJΔaglQ** | pBJ114-*aglQ* in-frame deletion |  |
| **pEH51** | pBJ114 bearing *aglQ-mCherry* fusion for double homologous recombination at native site | This work |
| **pDK21** | pBJ114 –*gltC* in-frame deletion | This work |
| **pDK22** | pBJ114 –*gltA* in-frame deletion | This work |
| **pDK23** | pBJ114 –*cglC* in-frame deletion | This work |
| **pDK25** | pBJ114 –*gltB* in-frame deletion | This work |
| **pNG020** | pBJ114 –*oar* in-frame deletion | This work |
| **pDK110** | pSW105 –P*_pilA_*-*cglC* | This work |
| **pDK111** | pSW105 –P*_pilA_*-*gltB* | This work |
| **pDK112** | pSW105 –P*_pilA_*-*gltA* | This work |
| **pDK113** | pSW105 –P*_pilA_*-*gltC* | This work |
| **pBJA1** | pET 45b^+^ -*cglC* | This work |
| **pBJA10** | pET 24b^+^ -*gltB* | This work |
| **pBJA3** | pET 45b^+^ -*gltA* | This work |
| **pBJA9** | pET 24b^+^ -*gltC* | This work |
| **pBJA26** | pMal-c2x –*cglC* | This work |
| **pBJA27** | pMal-c2x –*gltB* | This work |
| **pBJA28** | pMal-c2x –*gltA* | This work |
| **pBJA29** | pGEX-4T1 –*gltB* | This work |
| **pBJA38** | pBJ114 –*agmP* in-frame deletion | This work |
| **pBJA31** | pSWU30 – P*_nat_-cglC* | This work |
| **pBJA32** | pSWU30 – P*_nat_-gltB* | This work |
| **pBJA33** | pSWU30 – P*_nat_-gltA* | This work |
| **pBJA34** | pSWU30 – P*_nat_-gltC* | This work |
| **pBJA35** | pSWU30 – P*_nat_-gltB-mCherry* | This work |
| **pBJA36** | pSWU30 – P*_nat_-gltA-mCherry* | This work |
| **pBJA37** | pSWU30 – P*_nat_-gltC-mCherry* | This work |

**Supplemental references**

1. Julien B, Kaiser AD, Garza A (2000) Spatial control of cell differentiation in *Myxococcus xanthus*. Proc Natl Acad Sci USA 97: 9098-9103.

2. Jakovljevic V, Leonardy S, Hoppert M, Søgaard-Andersen L (2008) PilB and PilT are ATPases acting antagonistically in type IV pilus function in *Myxococcus xanthus*. J Bacteriol 190: 2411-2421.

3. Leonardy S, Miertzschke M, Bulyha I, Sperling E, Wittinghofer A, Søgaard-Andersen L (2010) Regulation of dynamic polarity switching in bacteria by a Ras-like G-protein and its cognate GAP. EMBO J 29: 2276-2289.

4. Sun MZ, Wartel M, Cascales E, Shaevitz JW, Mignot T (2011) Motor-driven intracellular transport powers bacterial gliding motility. Proc Natl Acad Sci USA 108: 7559-7564.
